# Sampling effects and measurement overlap can bias the inference of neuronal avalanches

**DOI:** 10.1371/journal.pcbi.1010678

**Published:** 2022-11-29

**Authors:** Joao Pinheiro Neto, F. Paul Spitzner, Viola Priesemann

**Affiliations:** 1 Max Planck Institute for Dynamics and Self-Organization, Göttingen, Germany; 2 Bernstein Center for Computational Neuroscience, Göttingen, Germany; 3 Georg-August University Göttingen, Göttingen, Germany; University of Freiburg, GERMANY

## Abstract

To date, it is still impossible to sample the entire mammalian brain with single-neuron precision. This forces one to either use spikes (focusing on few neurons) or to use coarse-sampled activity (averaging over many neurons, e.g. LFP). Naturally, the sampling technique impacts inference about collective properties. Here, we emulate both sampling techniques on a simple spiking model to quantify how they alter observed correlations and signatures of criticality. We describe a general effect: when the inter-electrode distance is small, electrodes sample overlapping regions in space, which increases the correlation between the signals. For coarse-sampled activity, this can produce power-law distributions even for non-critical systems. In contrast, spike recordings do not suffer this particular bias and underlying dynamics can be identified. This may resolve why coarse measures and spikes have produced contradicting results in the past.

## Author summary

The criticality hypothesis associates functional benefits with neuronal systems that operate in a dynamic state at a critical point. A common way to probe the dynamic state of a neuronal systems is measuring characteristics of so-called avalanches—distinct cascades of neuronal activity that are separated in time. For example, the probability distribution of the avalanche size will resemble a power-law if a neuronal system is critical. Thus, power-law distributions have become a common indicator for critical dynamics.

Here, we use simple models and numeric simulations to show that not only the dynamic state of a system has an impact on avalanche distributions. Also aspects that are only related to the sampling of the system (such as inter-electrode distance) or the way avalanches are calculated (such as thresholding and time binning) can shape avalanche distributions. On a mechanistic level we find that, if electrodes record spatially overlapping regions, the signals of electrodes may be *spuriously* correlated; multiple electrodes might pick up activity from the same neuron. Subsequently, when avalanches are inferred, such a measurement overlap can produce power-law distributions even if the underlying system is not critical.

## 1 Introduction

For more than two decades, it has been argued that the cortex might operate at a critical point [1–7]. The criticality hypothesis states that by operating at a critical point, neuronal networks could benefit from optimal information-processing properties. Properties maximized at criticality include the correlation length [8], the autocorrelation time [6], the dynamic range [9, 10] and the richness of spatio-temporal patterns [11, 12].

Evidence for criticality in the brain often derives from measurements of *neuronal avalanches*. Neuronal avalanches are cascades of neuronal activity that spread in space and time. If a system is critical, the probability distribution of avalanche size *p*(*S*) follows a power law *p*(*S*) ∼ *S*^−*α*^ [8, 13]. Such power-law distributions have been observed repeatedly in experiments since they were first reported by Beggs & Plenz in 2003 [1].

However, not all experiments have produced power laws and the criticality hypothesis remains controversial. It turns out that results for cortical recordings *in vivo* differ systematically:

Studies that use what we here call *coarse-sampled* activity typically produce power-law distributions [1, 14–23]. In contrast, studies that use *sub-sampled* activity typically do not [16, 24–28]. Coarse-sampled activity include LFP, M/EEG, fMRI and potentially calcium imaging, while sub-sampled activity is front-most spike recordings. We hypothesize that the apparent contradiction between coarse-sampled (LFP-like) data and sub-sampled (spike) data can be explained by the differences in the recording and analysis procedures.

In general, the analysis of neuronal avalanches is not straightforward. In order to obtain avalanches, one needs to define discrete events. While spikes are discrete events by nature, a coarse-sampled signal has to be converted into a binary form. This conversion hinges on thresholding the signal, which can be problematic [29–32]. Furthermore, events have to be grouped into avalanches, and this grouping is typically not unique [24]. As a result, avalanche-size distributions depend on the choice of the threshold and temporal binning [1, 33].

In this work, we show how thresholding and temporal binning interact with a commonly ignored effect [16, 34]. Under coarse-sampling, neighboring electrodes may share the same field-of-view. This creates a distance-dependent *measurement overlap* so that the activity that is recorded at different electrodes may show *spurious correlations*, even if the underlying spiking activity is fully uncorrelated. We show that the inter-electrode distance may therefore impact avalanche-size distributions more severely than the underlying neuronal activity.

In this numeric study, we explore the role of the recording and analysis procedures on a locally-connected network of simple binary neurons. Focusing on avalanche distributions, we compare apparent signs of criticality under sub-sampling versus coarse-sampling. To that end, we vary the distance to criticality of the underlying system over a wide range, from uncorrelated (Poisson) to highly-correlated (critical) dynamics. We then employ a typical analysis pipeline to derive signatures of criticality and study how results depend on electrode distance and temporal binning.

## 2 Results

The aim of this study is to understand *how the sampling of neural activity* affects the inference of the underlying collective dynamics. This requires us to be able to precisely set the underlying dynamics. Therefore, we use the established branching model [35], which neglects many biophysical details, but it allows us to precisely tune the dynamics and to set the distance to criticality.

To study sampling effects, we use a two-level setup inspired by [34]: an underlying network model, on which activity is then *sampled* with a grid of 8 × 8 virtual electrodes. Where possible, parameters of the model, the sampling and the analysis are motivated by values from experiments (see Methods).

In order to evaluate sampling effects, we want to *precisely* set the underlying dynamics. The branching model meets this requirement and is well understood analytically [11, 27, 34–36]. Inspired by biological neuronal networks, we simulate the branching dynamics on a 2D topology with *N*_N_ = 160 000 neurons where each neuron is connected to *K* ≈ 1000 local neighbors. To emphasize the locality, the synaptic strength of connections decays with the distance *d*_N_ between neurons. For a detailed comparison with different topologies, see the Supplemental Information (Fig A in S1 Text).

### 2.1 Avalanches are extracted differently under coarse-sampling and sub-sampling

At each electrode, we sample both the spiking activity of the closest neuron (sub-sampling) and a spatially averaged signal that emulates LFP-like coarse-sampling.

**Fig 1:**
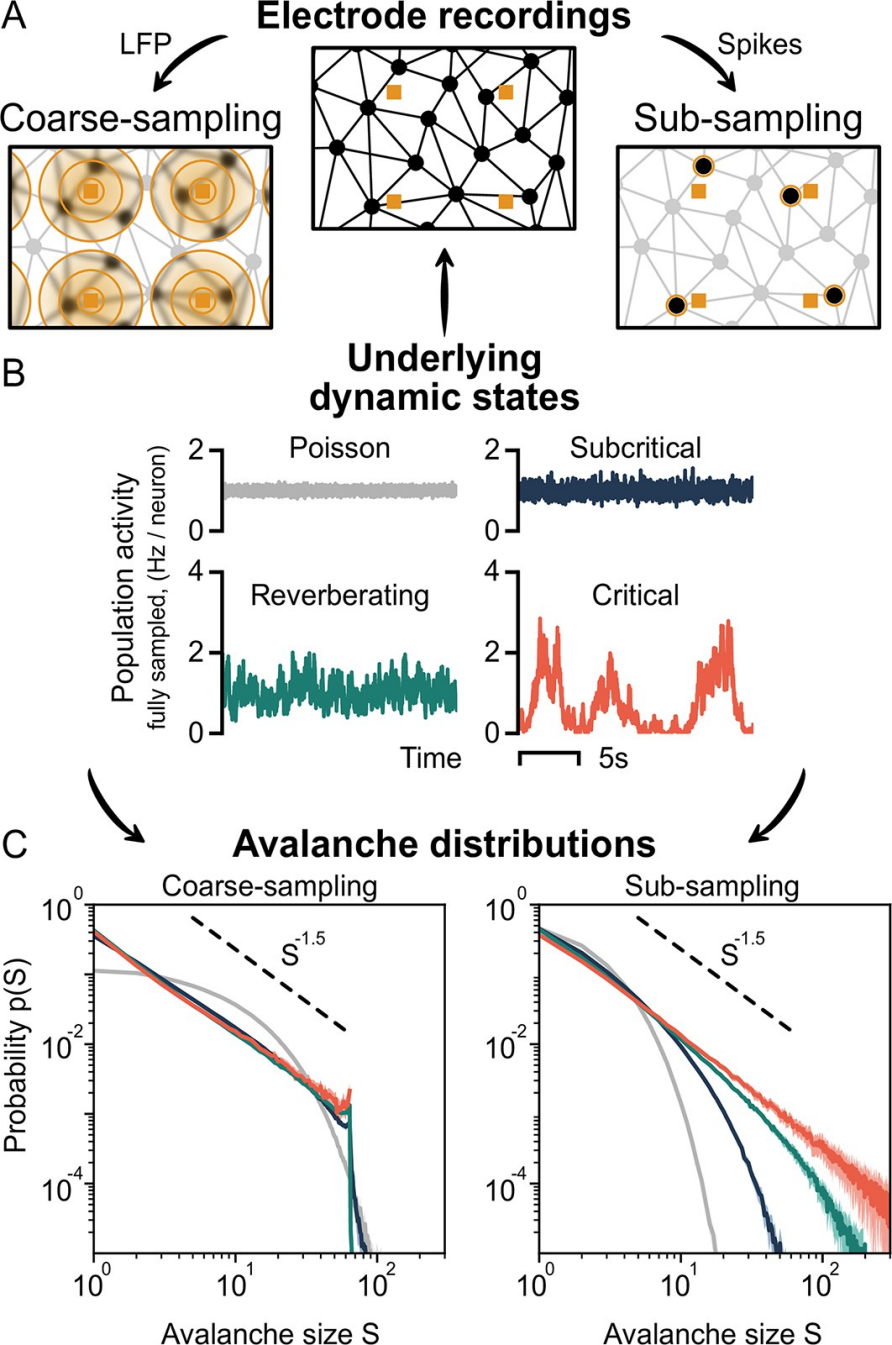
Sampling affects the assessment of dynamic states from neuronal avalanches. **A**: Representation of the sampling process of neurons (black circles) using electrodes (orange squares). Under coarse-sampling (e.g. LFP), activity is measured as a weighted average in the electrode’s vicinity. Under sub-sampling (spikes), activity is measured from few individual neurons. **B**: Fully sampled population activity of the neuronal network, for states with varying intrinsic timescales *τ*: Poisson (${\hat{\tau }}_{\text{psn}}\approx 0\;\text{ms}$), subcritical (${\hat{\tau }}_{\text{sub}}\approx 19\;\text{ms}$), reverberating (${\hat{\tau }}_{\text{rev}}\approx 98\;\text{ms}$) and critical (${\hat{\tau }}_{\text{crit}}\approx 1.6\;\text{s}$). **C**: Avalanche-size distribution *p*(*S*) for coarse-sampled (left) and sub-sampled (right) activity. Sub-sampling allows for separating the different states, whereas coarse-sampling leads to *p*(*S*) ∼ *S*^−*α*^ for all states except Poisson. **Parameters**: Electrode contribution *γ* = 1, inter-electrode distance *d*_E_ = 400 μm and time-bin size Δ*t* = 8 ms.

Both *coarse-sampling* and *sub-sampling* are sketched in Fig 1A: For coarse-sampling (left), the signal from each electrode channel is composed of varying contributions (orange circles) of all surrounding neurons. The contribution of a particular spike from neuron *i* to electrode *k* decays as $1/d_{ik}^{\gamma }$ with the neuron-to-electrode distance *d*_*ik*_ and electrode contribution *γ* = 1. In contrast, if spike detection is applied (Fig 1A, right), each electrode signal captures the spiking activity of few individual neurons (highlighted circles).

In order to focus on the key mechanistic differences between the two sampling approaches, we keep the two models as simple as possible. (This also matches the simple underlying dynamics, for which we can precisely set the distance to criticality). However, especially for coarse-sampling, this yields a rather crude approximation: More realistic, biophysically detailed LFP models would yield much more complex distance dependencies, which are an open field of research [37–40]. Our chosen electrode-contribution of *γ* = 1 assumes a large field of view, which implies the strongest possible measurement overlap to showcase the coarse-sampling effect. As this is an important assumption, we consider electrodes with a smaller field of view in Sec. 2.5 and provide an extended discussion in the Supplemental Information (Fig B in S1 Text).

**Fig 2:**
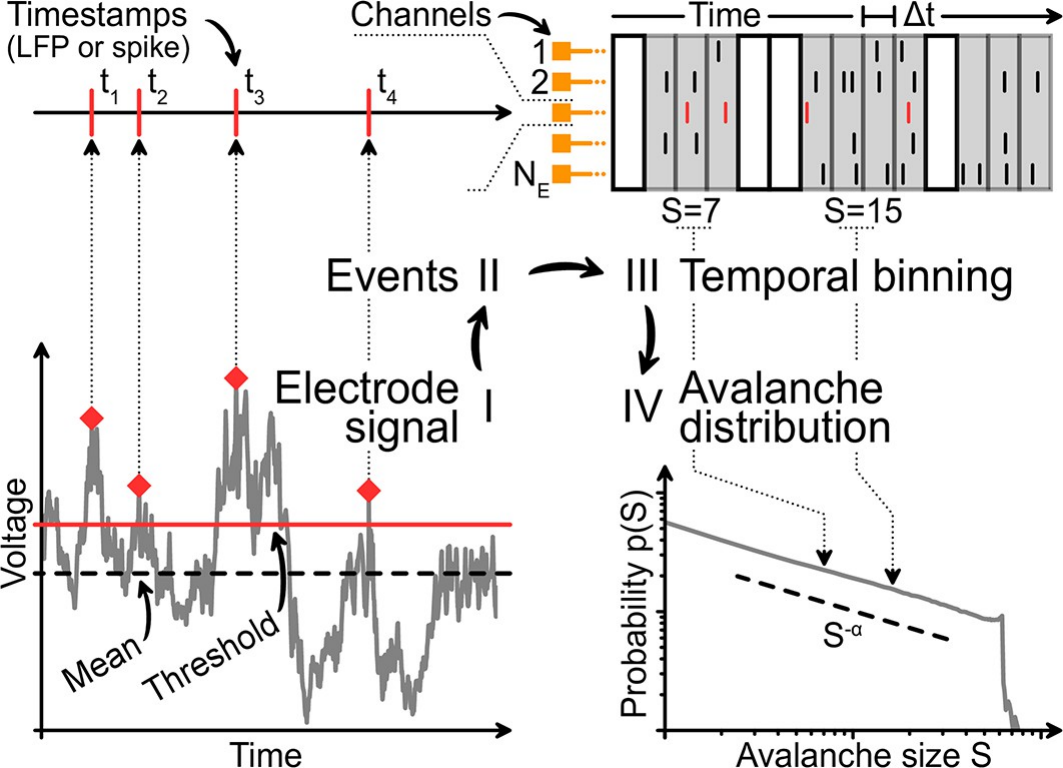
Analysis pipeline for avalanches from sampled data. **I**: Under coarse-sampling (LFP-like), the recording is demeaned and thresholded. **II**: The timestamps of events are extracted. Under sub-sampling (spikes), timestamps are obtained directly. **III**: Events from all channels are binned with time-bin size Δ*t* and summed. The size *S* of each neuronal avalanche is calculated. **IV**: The probability of an avalanche size is given by the (normalized) count of its occurrences throughout the recording.

To test both recording types for criticality, we apply the standard analysis that provides a probability distribution *p*(*S*) of the *avalanche size* *S*: In theory, an avalanche describes a cascade of activity where individual units—here neurons—are consecutively and causally activated. Each activation is called an event. The avalanche size is then the total number of events in the time between the first and the last activation. A power law in the size distribution of these avalanches is a hallmark of criticality [6]. In practice, the actual size of an avalanche is hard to determine because individual avalanches are not clearly separated in time; the coarse-sampled signal is continuous-valued and describes the local population. In order to extract binary events for the avalanche analysis (Fig 2), the signal has to be thresholded—which is not necessary for spike recordings, where binary events are inherently present as timestamps.

### 2.2 The branching parameter *m* sets the distance to criticality

In order to compare apparent signatures of criticality with the true, underlying dynamics, we first give some intuition about the branching model. The *branching parameter* *m* quantifies the probability of *postsynaptic activations*, or in other words, how many subsequent spikes are caused (on average) by a single spike. With increasing *m* → 1, a single spike triggers increasingly long cascades of activity. These cascades determine the timescale over which fluctuations occur in the population activity—this *intrinsic timescale* *τ* describes the dynamic state of the system and its distance to criticality.

The intrinsic timescale can be analytically related to the branching parameter by *τ* ∼ −1/ln(*m*). As *m* → 1, *τ* → ∞ and the population activity becomes “bursty”. We illustrate this in Fig 1B and Table 1: For Poisson-like dynamics (*m* ≈ 0), the intrinsic timescale is zero (${\hat{\tau }}_{\text{psn}}\approx 0\;\text{ms}$) and the activity between neurons is uncorrelated. As the distance to criticality becomes smaller (*m* → 1), the intrinsic timescale becomes larger (${\hat{\tau }}_{\text{sub}}\approx 19\;\text{ms}$, ${\hat{\tau }}_{\text{rev}}\approx 98\;\text{ms}$, ${\hat{\tau }}_{\text{crit}}\approx 1.6\;\text{s}$), fluctuations become stronger, and the spiking activity becomes more and more correlated in space and time. Apart from critical dynamics, of particular interest in the above list is the “reverberating regime”: For practical reasons, we assign a specific value of *m* (Table 1), which represents typical values observed *in vivo* [41, 42]. However, this choice is meant as a representation for a regime that is close-to-critical, but not directly at the critical point. In this regime, many of the benefits of criticality emerge, while the system can maintain a safety-margin from instability [41].

**Table 1:** Parameters and intrinsic timescales of dynamic states. All combinations of branching parameter *m* and per-neuron drive *h* result in a stationary activity of 1 Hz per neuron. Due to the recurrent topology, it is more appropriate to consider the measured autocorrelation time $\hat{\tau }$ rather than the analytic timescale *τ*.

State name	*m*	$\hat{\tau }$ (measured)	$\tau =\frac{-2\;\text{ms}}{\text{ln}\;m}$	*h*
Poisson	0.0	0.1 ± 0.1 ms	0.0 ms	2 × 10^−3^
Subcritical	0.9	18.96 ± 0.09 ms	18.9 ms	2 × 10^−4^
Reverberating	0.98	98.3 ± 1.0 ms	98.9 ms	4 × 10^−5^
Critical	0.999	1.58 ± 0.12 s	1.99 s	2 × 10^−6^

### 2.3 Coarse-sampling can cloud differences between dynamic states

Irrespective of the applied sampling, the inferred avalanche distribution *should* represent the true dynamic state of the system.

However, under coarse-sampling (Fig 1C, left), the avalanche-size distributions of the subcritical, reverberating and critical state are virtually indistinguishable. Intriguingly, all three show a power law. The observed exponent *α* = 1.5 is associated with a critical branching process. Only the uncorrelated (Poisson-like) dynamics produce a non-power-law decay of the avalanche-size distribution.

Under sub-sampling (Fig 1C, right), each dynamic state produces a unique avalanche-size distribution. Only the critical state, with the longest intrinsic timescale, produces the characteristic power law. Even the close-to-critical, reverberating regime is clearly distinguishable and features a “subcritical decay” of *p*(*S*).

### 2.4 Measurement overlap causes spurious correlations

Why are the avalanche-size distributions of different dynamic states hard to distinguish under coarse-sampling? The answer is hidden within the cascade of steps involved in the recording and analysis procedure. Here, we separate the impact of the involved processing steps. Most importantly, we discuss the consequences of *measurement overlap*—which we identify as a key explanation for the ambiguity of the distributions under coarse-sampling.

In order to obtain discrete events from the continuous time series for the avalanche analysis, each electrode signal is filtered and thresholded, binned with a chosen time-bin size Δ*t* and, subsequently, the events from all channels are stacked. This procedure is problematic because **(i)** electrode proximity adds spatial correlations, **(ii)** temporal binning adds temporal correlations, and **(iii)** thresholding adds various types of bias [29–31].

**Fig 3:**
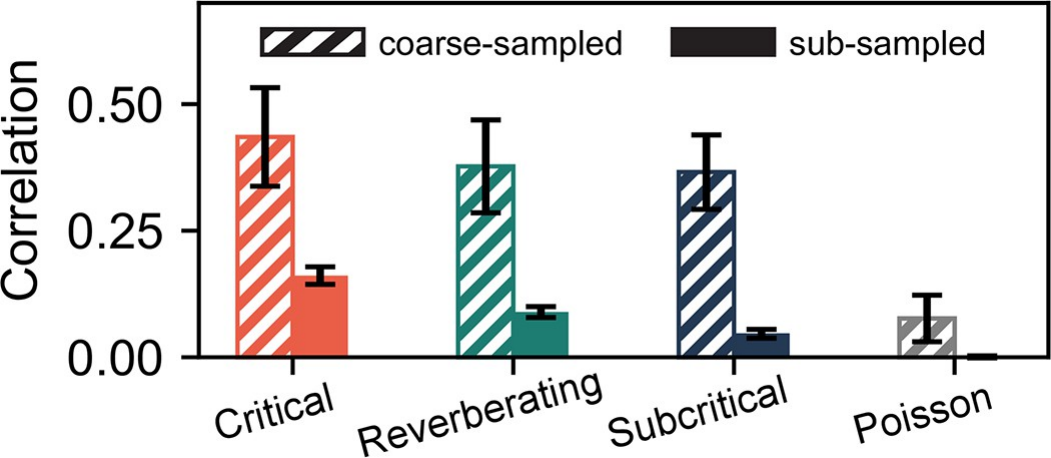
Coarse-sampling leads to greater correlations than sub-sampling. Pearson correlation coefficient between the signals of two adjacent electrodes for the different dynamic states. Even for independent (uncorrelated) Poisson activity, measured correlations under coarse-sampling are non-zero. **Parameters**: Electrode contribution *γ* = 1, inter-electrode distance *d*_E_ = 400 μm and time-bin size Δ*t* = 8 ms.

As a result of the involved analysis of coarse-sampled data, spurious correlations are introduced that are not present in sub-sampled data. We showcase this effect in Fig 3, where the Pearson correlation coefficient between two virtual electrodes is compared for both the (thresholded and binned) coarse-sampled and sub-sampled activity. For the same parameters and dynamic state, coarse-sampling leads to larger correlations than sub-sampling.

Depending on the sensitivity and distance between electrodes, multiple electrodes might record activity from the same neuron. This **measurement overlap** (or volume conduction effect) increases the spatial correlations between electrodes—and because the signals from multiple electrode channels are combined in the analysis, correlations can originate from measurement overlap alone.

### 2.5 Measurement overlap depends on electrodes’ field of view

**Fig 4:**
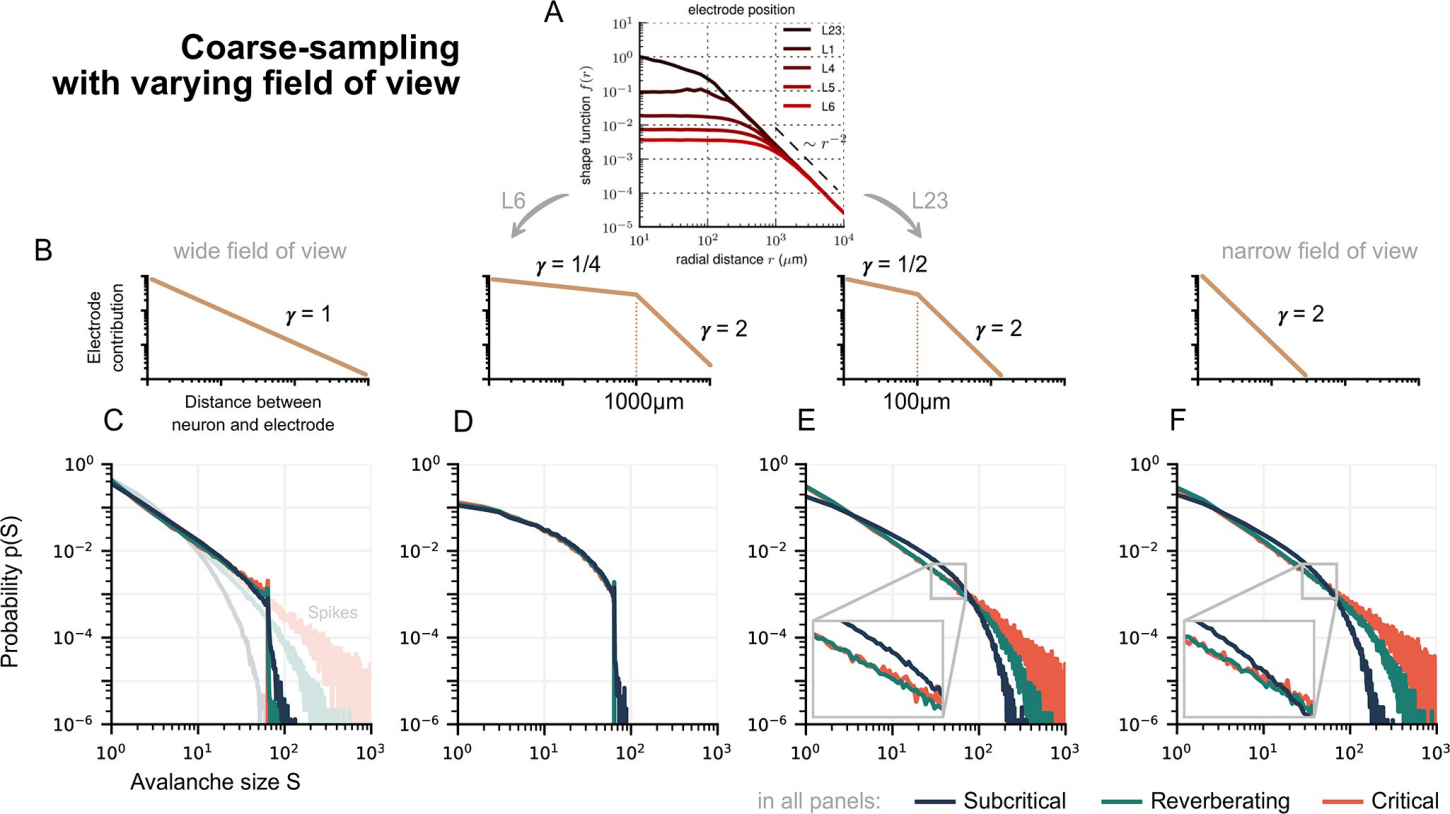
The signal of an extracellular neuronal recording depends on neuronal morphologies, tissue filtering, and other factors, which all impact the coarse-sampling effect. In effect, an important factor is the distance of the neuron to the electrode. Here, we show how the distance-dependence, with which a neuron’s activity contributes to an electrode, determines the collapse of avalanche distributions. **A**: Biophysically plausible distance dependence of LFP, reproduced from [38]. **B**: Sketch of a neuron’s contribution to an electrode at distance *d*_*ik*_, as motivated by (A). The decay exponent *γ* characterizes the field of view. **C–F**: Avalanche-size distribution *p*(*S*) for coarse-sampling with the sketched electrode contributions. **C, D**: With a wide-field of view, distributions are hardly distinguishable between dynamic states. In contrast, for spiking activity the differences are clear (light shades in C). **E, F**: With a narrower field of view, distributions do not fully collapse on top of each other, but differences between reverberating and critical dynamics remain hard to identify. **Parameters**: Inter-electrode distance *d*_E_ = 400 μm and time-bin size Δ*t* = 8 ms. Other parameter combinations in Fig B in S1 Text.

The amount of measurement overlap between electrodes is determined effectively by the electrodes’ field of view, thus the distance dependence with which a neuron’s activity *s*_*i*_ contributes to the electrode signal *V*_*k*_ (Fig 4). We consider electrode signals $V_{k}\left(t\right)=\sum_{i}^{N_{N}}s_{i}\left(t\right)/d_{ik}^{\gamma }$, where the exponent *γ* indicates how narrow (*γ* = 2) or wide (*γ* = 1) the field of view is. Note that realistic distance dependencies are more complex and depend on many factors, such as neuron morphology and tissue filtering [37–40].

We find that the collapse of avalanche-size distributions from different dynamic states is strongest when the field of view is wide—i.e. if there is stronger measurement overlap. In that case, coarse-sampled distributions are hardly distinguishable (Fig 4C and 4D). For a narrow field of view, distributions are still hard to distinguish but do not fully collapse (Fig 4E and 4F).

In order to study the impact of inter-electrode distance and temporal binning, in the following we focus on the wide field of view (*γ* = 1) where the avalanche collapse is most pronounced.

### 2.6 The effect of inter-electrode distance

**Fig 5:**
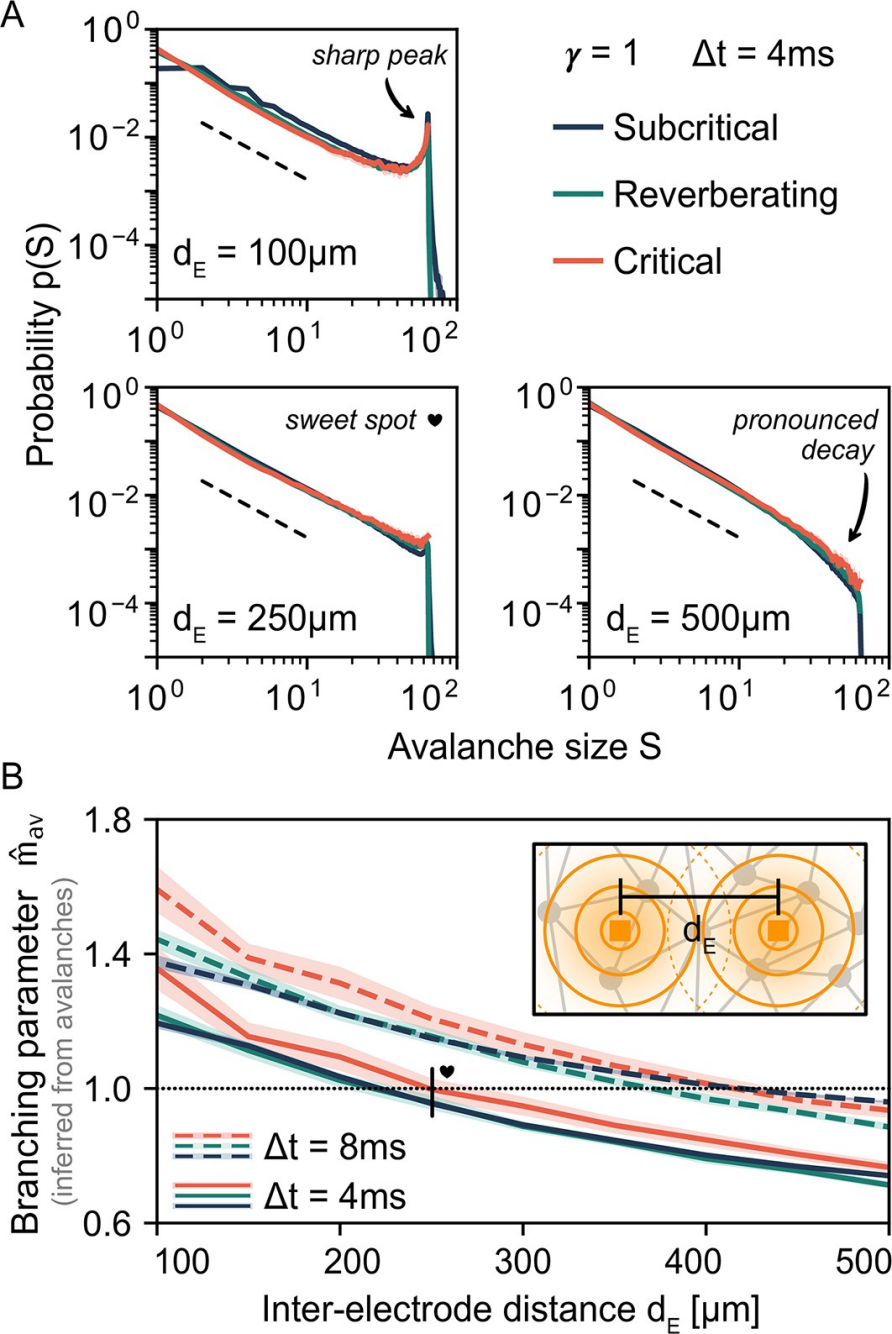
Under coarse-sampling, apparent dynamics depend on the inter-electrode distance *d*_E_. **A**: For small distances (*d*_E_ = 100 μm), the avalanche-size distribution *p*(*S*) indicates (apparent) supercritical dynamics: *p*(*S*) ∼ *S*^−*α*^ with a *sharp peak* near the electrode number *N*_E_ = 64. For large distances (*d*_E_ = 500 μm), *p*(*S*) indicates subcritical dynamics: *p*(*S*) ∼ *S*^−*α*^ with a *pronounced decay* already for *S* < *N*_E_. There exists a *sweet-spot* value (*d*_E_ = 250 μm) for which *p*(*S*) indicates critical dynamics: *p*(*S*) ∼ *S*^−*α*^ until the the cut-off is reached at *S* = *N*_E_. The particular sweet-spot value of *d*_E_ depends on time-bin size (here, Δ*t* = 4 ms). As a guide to the eye, dashed lines indicate *S*^−1.5^. **B**: The inferred branching parameter ${\hat{m}}_{\text{av}}$ is also biased by *d*_E_ when estimated from neuronal avalanches. Apparent criticality (${\hat{m}}_{\text{av}}\approx 1$, dotted line) is obtained with *d*_E_ = 250 μm and Δ*t* = 4 ms but also with *d*_E_ = 400 μm and Δ*t* = 8 ms. **B, Inset**: representation of the measurement overlap between neighboring electrodes; when electrodes are placed close to each other, spurious correlations are introduced.

Similar to the field of view of electrodes, avalanche-size distributions under coarse-sampling depend on the inter-electrode distance *d*_E_ (Fig 5A). For small inter-electrode distances, the overlap is strong. Thus, the spatial correlations are strong. Strong correlations manifest themselves in *larger* avalanches. However, under coarse-sampling the maximal observed size *S* of an avalanche is in general limited by the number of electrodes *N*_E_ [34] (cf. Fig B in S1 Text). This limit due to *N*_E_ manifests as a sharp cut-off and—in combination with spurious measurement correlations due to *d*_E_—can shape the probability distribution. In the following, we show that these factors can be more dominant than the actual underlying dynamics.

In theory, supercritical dynamics are characterized by a *sharp peak* in the avalanche distribution at *S* = *N*_E_. Independent of the underlying dynamics, such a peak can originate from small electrode distances (Fig 5A, *d*_E_ = 100 μm): Avalanches are likely to span the small area covered by the electrode array. Furthermore, due to strong measurement overlap, individual events of the avalanche may contribute strongly to multiple electrodes.

Subcritical dynamics are characterized by a *pronounced decay* already for *S* < *N*_E_. Independent of the underlying dynamics, such a decay can originate from large electrode distances (Fig 5A, *d*_E_ = 500 μm): Locally propagating avalanches are unlikely to span the large area covered by the electrode array. Furthermore, due to the weaker measurement overlap, individual events of the avalanche may contribute strongly to one electrode (or to multiple electrodes but only weakly).

Consequently, there exists a *sweet-spot* value of the inter-electrode distance *d*_E_ for which *p*(*S*) appears convincingly critical (Fig 5A, *d*_E_ = 250 μm): a power law *p*(*S*)∼*S*^−*α*^ spans all sizes up to the cut-off at *S* = *N*_E_. However, the dependence on the underlying dynamic state is minimal.

**Fig 6:**
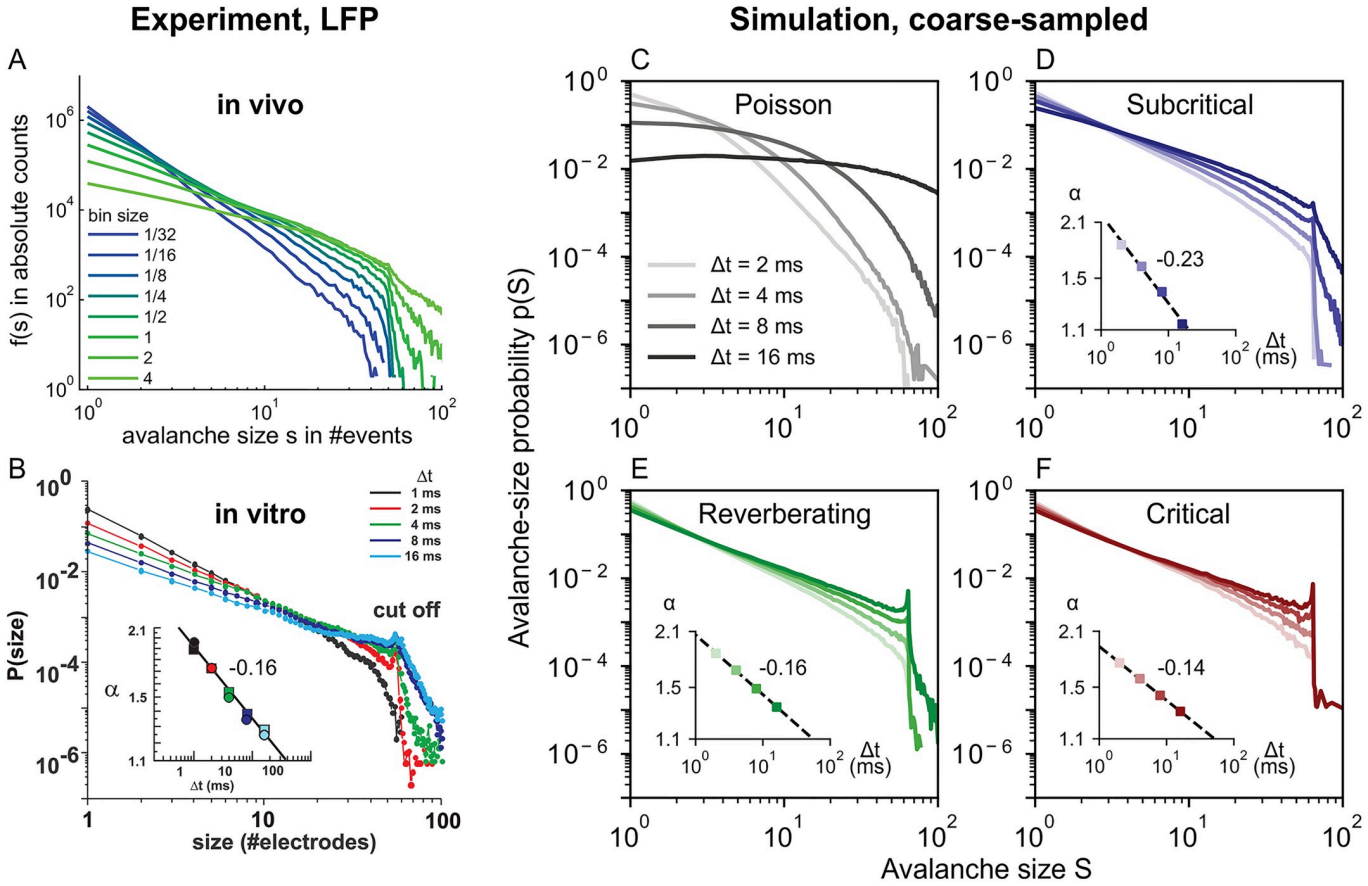
In vivo and in vitro avalanche-size distributions *p*(*S*) from LFP depend on time-bin size Δ*t*. Experimental LFP results are reproduced by many dynamics states of coarse-sampled simulations. **A**: Experimental *in vivo* results (LFP, human) from an array of 60 electrodes, adapted from [43]. **B**: Experimental *in vitro* results (LFP, culture) from an array with 60 electrodes, adapted from [1]. **C–F**: Simulation results from an array of 64 virtual electrodes and varying dynamic states, with time-bin sizes between 2 ms ≤ Δ*t* ≤ 16 ms, *γ* = 1 and *d*_E_ = 400 μm. Subcritical, reverberating and critical dynamics produce approximate power-law distributions with bin-size-dependent exponents *α*. **Insets**: Log-Log plot, distributions are fitted to *p*(*S*) ∼ *S*^−*α*^, fit range *S* ≤ 50. The magnitude of *α* decreases as Δ*t*^−*β*^ with −*β* indicated next to the insets, cf. Table 2.

**Fig 7:**
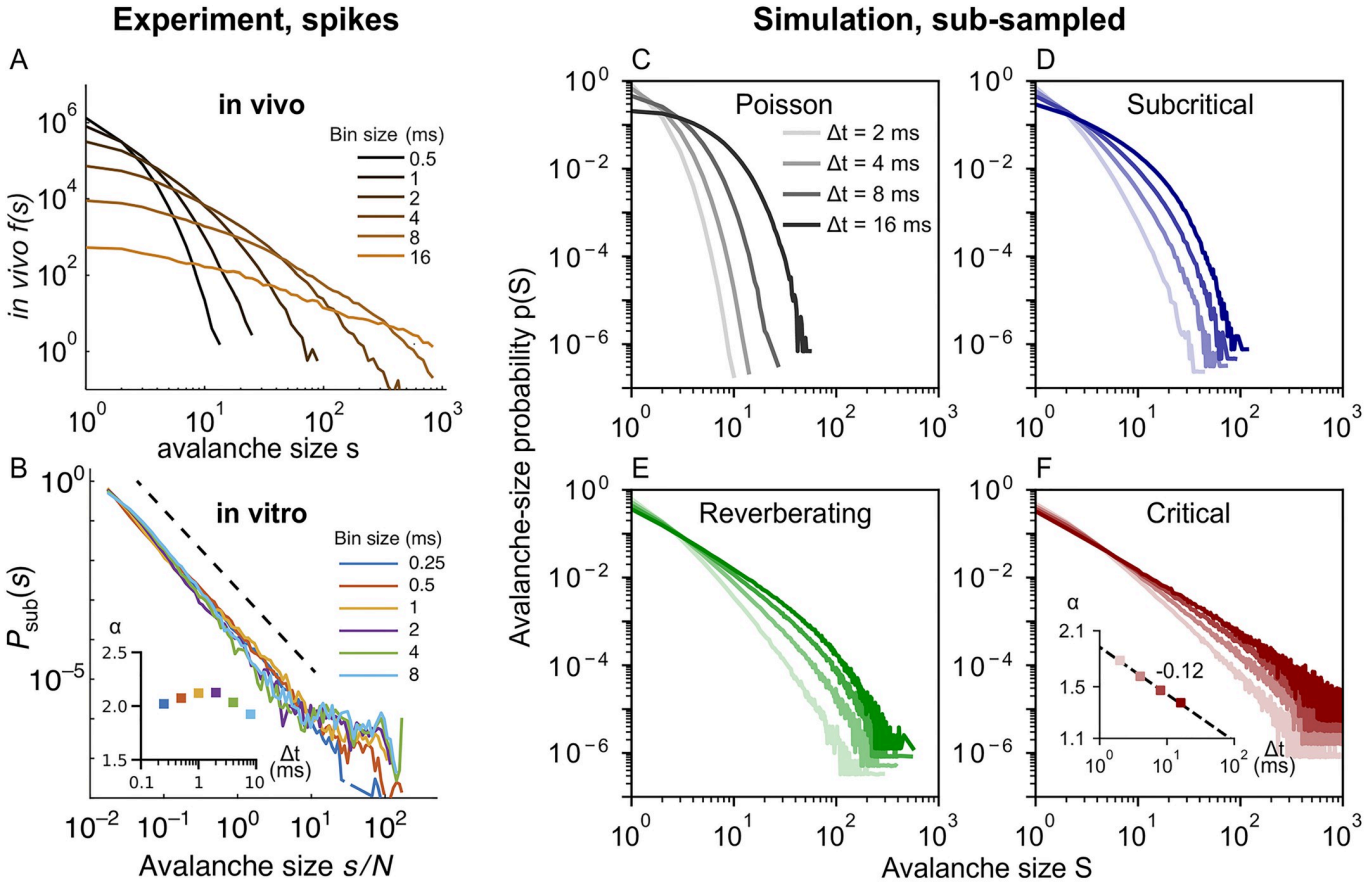
*In vivo* avalanche-size distributions *p*(*S*) from spikes depend on time-bin size Δ*t*. ***In vivo* results from spikes are reproduced by sub-sampled simulations of subcritical to reverberating dynamics**. Neither spike experiments nor sub-sampled simulations show the cut-off that is characteristic under coarse-sampling. **A**: Experimental *in vivo* results (spikes, awake monkey) from an array of 16 electrodes, adapted from [24]. The pronounced decay and the dependence on bin size indicate subcritical dynamics. **B**: Experimental *in vitro* results (spikes, culture DIV 34) from an array with 59 electrodes, adapted from [44]. Avalanche-size distributions are largely independent of time-bin size and resemble a power law over four orders of magnitude. In combination, this indicates a separation of timescales and critical dynamics (or even super critical dynamics [45]). **B, Inset**: Log-Lin plot of fitted *α*, fit range *s*/*N* ≤ 5. **C–F**: Simulation for sub-sampling, analogous to Fig 6. Subcritical dynamics do not produce power-law distributions and are clearly distinguishable from critical dynamics. **F**: Only the (close-to) critical simulation produces power-law distributions. **F, Inset**: Log-Log plot of fitted *α*, fit range *S* ≤ 50. In contrast to the *in vitro* culture (in B), the simulation does not feature a separation of time scales (due to external drive and stationary activity), and therefore the slope shows a systematic bin-size dependence here.

Independently of the apparent dynamics, we observe the discussed cut-off at *S* = *N*_E_, which is also often seen in experiments (Fig 6). Note, however, that this cut-off only occurs under coarse-sampling (see again Fig 1C). When spikes are used instead (Fig 7), the same avalanche can reach an electrode repeatedly in quick succession—whereas such double-events are circumvented when thresholding at the population level. For more details see Fig B in S1 Text.

A further signature of criticality is obtained by inferring the branching parameter. If the inference is unbiased, the inferred $\hat{m}$ matches the underlying branching parameter *m*. We have developed a sub-sampling invariant estimator (based on the population activity inferred from spikes [27]), but $\hat{m}$ is traditionally inferred from avalanches. Then, ${\hat{m}}_{\text{av}}$ is defined as the average ratio of events between subsequent time bins in an avalanche, i.e. during non-zero activity [1, 33].

Obtaining ${\hat{m}}_{\text{av}}$ for different electrode distances results in a picture consistent with the one from avalanche-size distributions (Fig 5B). In general, the dependence on the electrode distance is stronger than the dependence on the underlying state. At the particular value of the inter-electrode distance where ${\hat{m}}_{\text{av}}=1$, the distributions appear critical. If ${\hat{m}}_{\text{av}}<1$ (${\hat{m}}_{\text{av}}>1$), the distributions appear subcritical (supercritical). Notably, the supercritical *m* > 1 corresponds to dynamics where activity increases indefinitely, which is not possible for systems of finite size and exposes ${\hat{m}}_{\text{av}}>1$ as an inference effect. More precisely, in case of our simulations, ${\hat{m}}_{\text{av}}$ suffers two sources of bias: firstly, the coarse-sampling bias that is rooted in the preceding avalanche analysis, and secondly the estimator assumes a pure branching process without specific topology or coalescence effects [36].

Concluding, because the probability distributions and the inferred branching parameter share the dependence on electrode distance, a wide range of dynamic states would be consistently misclassified—solely as a function of the inter-electrode distance.

### 2.7 Temporal binning determines scaling exponents

Apart from the inter-electrode distance, the choice of temporal discretization that underlies the analysis may alter avalanche-size distributions. This *time-bin size* Δ*t* varies from study to study and it can severely impact the observed distributions [1, 24, 43, 44]. With smaller bin sizes, avalanches tend to be separated into small clusters, whereas larger bin sizes tend to “glue” subsequent avalanches together [24]. Interestingly, this not only leads to larger avalanches, but specifically to *p*(*S*) ∼ *S*^−*α*^, where the exponent *α* increases systematically with bin size [1, 43]. Such a changing exponent is not expected for conventional systems that self-organize to criticality: Avalanches would be *separated in time*, and *α* should be fairly bin-size invariant for a large range of Δ*t* [24, 44, 46].

Our coarse-sampled model reproduces these characteristic experimental results (Fig 6). It also reproduces the previously reported scaling [1] of the exponent with bin size *α* ∼ Δ*t*^−*β*^ (cf. Fig 6 insets and Table 2). Except for the Poisson dynamics, all the model distributions show power laws. Moreover the distributions are strikingly similar, not just to the experimental results, but also to each other. This emphasizes how sensitive signs of criticality are to analysis parameters: All the shown dynamic states are consistent with the ubiquitous avalanche-size distributions that are observed in coarse-sampled experiments [45] (cf. Table A in S1 Text).

**Table 2:** Fitted exponents of *α* ∼ Δ*t*^−*β*^.

Dynamic state	*β*	
	*d*_E_ = 200 μm	*d*_E_ = 400 μm
in vitro (LFP) [1]	0.16 ± 0.01	
Critical (coarse)	0.113 ± 0.001	0.141 ± 0.001
Reverberating (coarse)	0.127 ± 0.003	0.156 ± 0.002
Subcritical (coarse)	0.159 ± 0.004	0.231 ± 0.016
Critical (spikes)	0.143 ± 0.010	0.123 ± 0.005

When spikes are used instead, power-law distributions only arise from critical dynamics. For comparison with the coarse-sampled results in Fig 6, we show avalanche-size distributions from experimental spike recordings and sub-sampled simulations in Fig 7.

In vivo spike recordings of awake animals produce distributions that feature a pronounced decay instead of power laws (Fig 7A). Interestingly, spike recordings of in vitro cultures often show power-laws and, here, even little-to-no bin-size dependence, which indicates a fairly good separation of timescales (Fig 7B). In this example, the power-law extends over several orders of magnitude, and the slope does not decrease systematically with the bin size. This indicates close-to-critical dynamics; the slight bump that represents an excess of very large avalanche, however, might also point to slight super-criticality [44, 45].

Considering our simulations of sub-sampling (Fig 7C–7F), we only observe approximate power laws if the model is (close-to) critical (Fig 7F). Note that in critical systems, the avalanche distribution should not change with bin size, and that here the bin-size dependence of the slope is caused by the finite system size and by the non-zero spike rate, which impede a proper separation of timescales. Nonetheless, in contrast to coarse-sampling, the avalanche distributions that stem from sub-sampled measures (spikes) allow us to clearly tell apart the underlying dynamic states from one another.

Overall, as our results on coarse-sampling have shown, different sources of bias—here the measurement overlap and the bin size—can perfectly outweigh each other. For instance, smaller electrode distances (that increase correlations) can be compensated by making the time-bin size smaller (which again decreases correlations). This was particularly evident in Fig 5B, where increasing *d*_E_ could be outweighed by increasing Δ*t* in order to obtain a particular value for the branching parameter *m*_av_. The same relationship was again visible in Fig 6C–6F: For the shown *d*_E_ = 400 μm (see also S1 Text for *d*_E_ = 200 μm), only Δ*t* = 8 ms results in *α* = 1.5—the correct exponent for the underlying dynamics. Since the electrode distance cannot be varied in most experiments, selecting anything but the one “lucky” Δ*t* will cause a bias.

## 3 Discussion

When inferring collective network dynamics from partially sampled systems, it is crucial to understand how the sampling biases the measured observables. Without this understanding, an elaborate analysis procedure—such as the one needed to study neuronal avalanches from coarse-sampled data—can result in a misclassification of the underlying dynamics.

We have shown that the analysis of neuronal avalanches based on (LFP-like) coarse-sampled data can cloud differences of avalanche distributions from systems with different spatio-temporal signatures. These signatures derive from underlying dynamic states that, in this work, range from subcritical to critical—a range over which the intrinsic timescale undergoes a hundred-fold increase. And yet, the resulting avalanche-size distributions can be ambiguous (Fig 1).

The ambiguity of neuronal avalanches partially originates from spurious correlations. We have demonstrated the generation of spurious correlations from two sampling- and processing mechanisms: measurement overlap (due to volume conduction) and temporal binning. Other studies found further mechanisms that can generate apparent power-law distributions by (purposely or accidentally) introducing correlations into the *observed* system. For instance, correlated input introduces temporal correlations already into the *underlying* system [47, 48]. Along with thresholding and low-pass frequency filtering—which add temporal correlations to the *observed* system [25, 49]—this creates a large space of variables that either depend on the system, sampling and processing, or a combination of both.

As our results focus on sampling and processing, we believe that the observed impact on avalanche-size distributions is general and model independent. We deliberately used simple models and confirmed that our results are robust to parameter and model changes: First, our model for coarse-sampling prioritizes simplicity over biophysical details—in order to be consistent with our simplified but well-controlled neuronal dynamics—but we checked that our results are consistent with different distance-dependencies or adding a cut-off (Figs B and C in S1 Text). Second, employing a more realistic topology causes no qualitative difference (Fig A in S1 Text). Third, as a proof of concept, we investigated the impact of measurement overlap in the 2D Ising model (Fig G in S1 Text). Even in such a fundamental model a measurement overlap can bias the assessment of criticality. Lastly, we investigated scaling relations (of avalanche size- and duration distributions) and found that under coarse-sampling, the inference is severely hindered (Fig F in S1 Text). Under sub-sampling, scaling relations hold but with a different collapse exponent than expected for our model. This is consistent with other recent work showing that sampling can affect the collapse exponent [50].

Despite these efforts, our work remains a mechanistic modeling study and we want to stress its limitations: Our virtual sampling did not account for neuron morphology nor the individual neuron’s connectivity profiles. As spikes are non-local events, both these aspects impact the sampling range of an electrode and the decay of e.g. an LFP signal [38, 40]. Sampling also depends on effects that occur prior to recording, such as possible filtering due to extracellular tissue [25, 51] or filtering due to neuron morphology [40, 52]. In particular, low-pass filtering can arise from synaptic dynamics or the propagation within dendrites [53]. Clearly, as high frequencies get stripped from the signal, this could attenuate deflections of the recorded time series. Because these deflections are central to the avalanche detection, low-pass filtering could, in principle, affect avalanche statistics. However, preliminary tests showed that our main result of overlapping distributions for different dynamics states remains intact when the raw time series are low-pass filtered (Fig E in S1 Text).

Our results seemingly contradict experimental studies that demonstrate that the avalanche analysis is sensitive to pharmacological manipulations such as anesthesia [18, 54–57]. Following a sufficient manipulation, a system’s dynamic state will change—which should be reflected by a visible difference of avalanche distributions. We showed that under coarse-sampling, the precise dynamic state could be misclassified. Whereas *subtle* differences between the avalanche distributions from different dynamic states are indeed visible (Fig 5), in general, they are clouded under coarse-sampling due to the measurement overlap. However, the smaller the measurement overlap becomes (e.g. through increasing the electrode-distance), the clearer the differences between dynamic states become (Fig B in S1 Text). In experiments the measurement overlap is unknown; it is also a priori unknown how strong a pharmacological perturbation is (relative to the equally unknown initial dynamic state) and how much coarse-sampling affects its inference. In modeling studies such as ours, these circumstances are well controlled—providing an explanation on a mechanistic level that can now be taken into consideration (and accounted for) when analyzing experimental data.

With our results on sampling effects, we can revisit the previous literature on neuronal avalanches. In Ref. [26] Ribeiro and colleagues show that “undersampling” biases avalanche distributions near criticality. In this case, undersampling was modeled by electrodes picking up a variable number of closest neurons. Here, we separated the effect of sub-sampling (electrodes cannot record all neurons) from coarse-sampling (electrodes record multiple neurons with distance-dependent contributions) and can add to previous results: In our model, we found that coarse-sampling clouds the differences between subcritical, reverberating, and critical dynamics; for *γ* = 1, the avalanche distributions always resemble power laws (Fig 4). Because of this ambiguity, the power-law distributions obtained ubiquitously from LFP, EEG, MEG and BOLD activity should be taken as evidence of neuronal activity with spatio-temporal correlations—but not necessarily of criticality proper; the coarse-sampling might hinder such a precise classification. In this regard, the interpretation of results from calcium imaging (which has a lower temporal resolution than electrode recordings) remains open (cf. Table A in S1 Text for an overview).

In contrast, a more precise classification seems possible when using spikes. If power-law distributions are observed from (sub-sampled) spiking activity, they do point to critical dynamics. For spiking activity, we even have mathematical tools to infer the precise underlying state in a sub-sampling-invariant manner that does not rely on avalanche distributions [27, 58]. However, not all spike recordings point to critical dynamics: Whereas in vitro recordings typically do produce power-law distributions [44, 59–61], extracellular spike recordings from awake animals typically do not [16, 18, 24, 62].

Lastly, our results might offer a solution to resolve an inconsistency between avalanche distributions that derive from spikes vs. LFP-like sampling: For experiments on awake animals, spike-based studies typically indicate subcritical dynamics. Although coarse measures typically produce power laws that indicate criticality, in this work we showed that they might cloud the difference between critical and subcritical dynamics. Consistent with both, a brain that operates in a *near*-critical regime—as opposed to a fixed dynamic state—could harness benefits associated with criticality while flexibly tuning its response properties [43, 63–69].

## 4 Methods

### 4.1 Model details

Our model is comprised of a two-level configuration, where a 2D network of *N*_N_ = 160000 spiking neurons is sampled by a square array of *N*_E_ = 8 × 8 virtual electrodes. Neurons are distributed randomly in space (with periodic boundary conditions) and, on average, nearest neighbors are *d*_N_ = 50 μm apart. While the model is inherently unit-less, it is more intuitive to assign some length scale—in our case the inter-neuron distance *d*_N_—to set that scale: all other size-dependent quantities can then be expressed in terms of the chosen *d*_N_. For instance, the linear system size *L* can be derived by realizing that the random placement of neurons corresponds to an ideal gas. It follows that $L=2\sqrt{N_{N}}\;d_{N}=4\text{cm}$ for uniformly distributed neurons. (For comparison, on a square lattice, the packing ratio would be higher and it is easy to see that the system size would be $\sqrt{N_{N}}\;d_{N}$.) Given the system size and neuron number, the overall neuronal density is *ρ* = 100/mm^2^. With our choice of parameters, the model matches typical experimental conditions in terms of inter-neuron distance and system size (see Table 3 for details). Whereas the apparent neuron density of *ρ* = 100/mm^2^ is on the lower end of literature values [70, 71], this parameter choice avoids boundary effects that can be particularly dominant near criticality due to the long spatial correlation. The implementation of the model in C++, and the python code used to analyze the data and generate the figures, are available online at https://github.com/Priesemann-Group/criticalavalanches.

**Table 3:** Values and descriptions of the model parameters.

Symbol	Value	Description
Δ*t*	2 − 16 ms	Time-bin size (duration) for temporal binning
Θ_*k*_	3	Activity threshold, in units of standard deviations of the time series of electrode *k*
δ*t*	2 ms	Simulation time step
*r*	1 Hz	Average spike rate
*N*_N_	1.6 × 10^5^	Number of neurons
*d*_N_	50 μm	Inter-neuron distance (measured between nearest neighbors)
*L*	4 cm	Linear system size
*ρ*	100/mm^2^	Neuronal density
*K*	1000	Average network degree (outgoing connections per neuron)
*d*_max_	1.78 mm	Connection length; all neurons within *d*_max_ are connected
*σ*	300 μm	Effective length of synaptic connections, sets the distance-dependence of the probabilities of recurrent activations
*N*_E_	8 × 8	Number of electrodes
*d*_E_	50 − 500 μm	Inter-electrode distance
$d_{E}^{*}$	10 μm	Dead-zone around each electrode (no neurons present)
*γ*	1	Decay exponent. Contributions of each spike to the coarse electrode signal scale as *V*(*d*) ∼ 1/*d*^*γ*^. See SI for results and discussion of different electrode contributions.

### 4.2 Topology

We consider a topology that enforces *local* spreading dynamics. Every neuron is connected to all of its neighbors within a threshold distance *d*_max_. The threshold is chosen so that on average *K* = 10^3^ outgoing connections are established per neuron. We thus seek the radius *d*_max_ of a disk whose area contains *K* neurons. Using the already known neuron density, we find $d_{\text{max}}=\sqrt{K/\pi \rho }\approx 1.78\;\text{mm}$. For every established connection, the probability of a recurrent activation decreases with increasing neuron distance. Depending on the particular distance *d*_*ij*_ between the two neurons *i* and *j*, the connection has a normalized weight $w_{ij}=e^{-d_{ij}^{2}/2\sigma^{2}}\;/\;\Omega_{i}$ (with normalization constant $\Omega_{i}=\sum_{j^{\prime }}e^{-d_{ij^{\prime }}^{2}/2\sigma^{2}}$). Our weight definition approximates the distance dependence of average synaptic strength. The parameter *σ* sets the *effective* distance over which connections can form (*d*_max_ is an upper limit for *σ* and mainly speeds up computation.) In the limit *σ* → ∞, the network is all-to-all connected. In the limit *σ* → 0, the network is completely disconnected. Therefore, the effective connection length *σ* enables us to fine tune *how local* the dynamic spreading of activity is. In our simulations, we choose *σ* = 6*d*_N_ = 300 μm. Thus, the overall reach is much shorter than *d*_max_ (*σ* ≈ 0.16 *d*_max_).

### 4.3 Dynamics

To model the dynamic spreading of activity, time is discretized to a chosen simulation time step, here *δt* = 2 ms, which is comparable to experimental evidence on synaptic transmission [72]. Our simulations run for 10^6^ time steps on an ensemble of 50 networks for each configuration (combination of parameters and dynamic state). This corresponds to ∼ 277 hours of recordings for each dynamic state.

The activity spreading is modeled using the dynamics of a branching process with external drive [27, 35]. At every time step *t*, each neuron *i* has a state *s*_*i*_(*t*) = 1 (spiking) or 0 (quiescent). If a neuron is spiking, it tries to activate its connected neighbors—so that they will spike in the next time step. All of these recurrent activations depend on the *branching parameter* *m*: Every attempted activation has a probability *p*_*ij*_ = *m* *w*_*ij*_ to succeed. (Note that the distance-dependent weights are normalized to 1 but the activation probabilities are normalized to *m*.) In addition to the possibility of being activated by its neighbors, each neuron has a probability *h* to spike spontaneously in the next time step. After spiking, a neuron is reset to quiescence in the next time step if it is not activated again.

Our model gives us full control over the dynamic state of the system—and its distance to criticality. The dynamic state is described by the *intrinsic timescale* *τ*. We can analytically calculate the intrinsic timescale *τ* = −*δt*/ln (*m*), where *δt* is the duration of each simulated time step. Note that *m*—the control parameter that *tunes the system*—is set on the neuron level while *τ* is a (collective) network property (that in turn allows us to deduce an *effective* *m*). As the system is pushed more towards criticality (by setting *m* → 1), the intrinsic timescale diverges *τ* → ∞.

For consistency, we measure the intrinsic timescale during simulations. To that end, the (fully sampled) population activity at each time step is given by the number of active neurons *A*(*t*) = ∑_*i*_ *s*_*i*_(*t*). A linear least-squares fit of the autoregressive relation *A*(*t* + 1) = *e*^−*δt*/*τ*^ *A*(*t*) + *N*_N_*h* over the full simulated time series yields an estimate $\hat{\tau }$ that describes each particular realization.

By adjusting the branching parameter *m* (setting the dynamic state) *and* the probability for spontaneous activations *h* (setting the drive), we control the distance to criticality *and* the average stationary activity. The activity is given by the *average spike rate* *r* = *h*/(*δt*(1 − *m*)) of the network. For all simulations, we fix the rate to *r* = 1Hz in order to avoid rate effects when comparing different states (see Table 1 for the list of parameter combinations). Note that, due to the non-zero drive *h* and the desired stationary activity, the model cannot be perfectly critical ($\hat{\tau }\to \infty$, see Table 1).

### 4.4 Coalescence compensation

With our probability-based update rules, it may happen that target neurons are simultaneously activated by multiple sources. This results in so-called *coalescence effects* that are particularly strong in our model due to the local activity spreading [36]. For instance, naively setting *m* = 1 (with *σ* = 300 μm) would result in an effective (measured) $\hat{m}\approx 0.98$, which has considerably different properties. Compared to e.g. *m* = 0.999 this would result in a 20-fold decrease in *τ*.

In order to compensate these coalescence effects, we apply a simple but effective fix: If an activation attempt is successful but the target neuron is already marked to spike in the next time step, another (quiescent) target is chosen. Because our implementation stores all the connected target neurons as a list sorted by their distance to the source, it is easy to activate the next neuron in that list. Thereby, the equivalent probability of the performed activation is as close to the originally attempted one as possible.

### 4.5 Virtual electrode recordings

Our simulations are designed to mimic sampling effects of electrodes in experimental approaches. To simulate sampling, we use the readout of *N*_E_ = 64 virtual electrodes that are placed in an 8 × 8 grid. Electrodes are separated by an inter-electrode distance that we specify in multiples of inter-neuron distance *d*_N_. It is kept constant for each simulation and we study the impact of the inter-electrode distance by repeated simulations spanning electrode distances between 1*d*_N_ = 50 μm and 10*d*_N_ = 500 μm. The electrodes are modeled to be point-like objects in space that have a small dead-zone of $d_{E}^{*}=d_{N}/5=10\;\mu m$ around their origin. Within the dead-zone, no signal can be recorded (in fact, we implement this by placing the electrodes first and the neurons second—and forbid neuron placements too close to electrodes).

Using this setup, we can apply sampling that emulates either the detection of spike times or LFP-like recordings. To model the detection of spike times, each electrode only observes the single neuron that is closest to it. Whenever this particular neurons spikes, the timestamp of the spike is recorded. All other neurons are neglected—and the dominant sampling effect is *sub-sampling*. On the other hand, to model LFP-like recordings, each electrode integrates the spiking of all neurons in the system. Contributions are strictly positive, matching the underlying branching dynamics (for more biophysically detailed LFP models, contributions would depend on neuron types and other factors). The contribution of a single spike, e.g. from neuron *i* to electrode *k*, decays as 1/*d*_*ik*_ with the neuron-to-electrode distance. (See Fig B in S1 Text for a detailed discussion of the qualitative impact of changing the distance dependence, e.g. to $1/d_{ik}^{2}$.) The total signal of the electrode at time *t* is then $V_{k}\left(t\right)=\sum_{i}^{N_{N}}s_{i}\left(t\right)/d_{ik}$. Diverging electrode signals are prevented by the forbidden zone around the electrodes. For such coarse-sampled activity, all neurons contribute to the signal and the contribution is weighted by their distance.

### 4.6 Avalanches

Taking into account all 64 electrodes, a new avalanche starts (by definition [1]) when there is at least one event (spike) in a time bin—given there was no event in the previous time bin (see Fig 2). An avalanche ends whenever an empty bin is observed (no event over the duration of the time bin). Hence, an avalanche persists for as long as every consecutive time bin contains at least one event—which is called the *avalanche duration* *D*. From here, it is easy to count the total number of events that were recorded across all electrodes and included time bins—which is called the *avalanche size* *S*. The number of occurrences of each avalanche size (or duration) are sorted into a histogram that describes the avalanche distribution.

### 4.7 Analysis of avalanches under coarse and sub-sampling

We analyze avalanche size distributions in a way that is as close to experimental practice as possible (see Fig 2). From the simulations described above, we obtain two outputs from each electrode: a) a list containing spike times of the single closest neuron and b) a time series of the integrated signal to which all neurons contributed.

In case of the (sub-sampled) spike times a), the spiking events are already present in binary form. Thus, to define a neural avalanche, the only required parameter is the size of the time bin Δ*t* (for instance, we may choose Δ*t* = 4 ms).

In case of the (coarse-sampled) time series b), binary events need to be extracted from the continuous electrode signal. The extraction of spike times from the continuous signal relies on a criterion to differentiate if the set of observed neurons is spiking or not—which is commonly realized by applying a threshold. (Note that now thresholding takes place on the electrode level, whereas previously, an event belonged to a single neuron.) Here, we obtain avalanches by thresholding as follows: First, all time series are frequency filtered to 0.1 Hz < *f* < 200 Hz. This demeans and smoothes the signal (and reflects common hardware-implemented filters of LFP recordings). Second, the mean and standard deviation of the full time series are computed for each electrode. The mean is virtually zero due to cutting low frequencies when band-pass filtering. Each electrode’s threshold is set to three standard deviations above the mean. Third, for every positive excursion of the time series (i.e. *V*_*k*_(*t*) > 0), we recorded the timestamp *t* = *t*_max_ of the maximum value of the excursion. An event was defined when *V*_*k*_(*t*_max_) was larger than the threshold Θ_*k*_ of three standard deviations of the (electrode-specific) time series. (Whenever the signal *passes the threshold*, the timestamps of all local maxima become candidates for the event; however, only the one largest maximum between two *crossings of the mean* assigns the final event-time.) Once the continuous signal of each electrode has been mapped to binary events with timestamps, the remaining analysis steps were the same for coarse-sampled and sub-sampled data. Last, avalanche size and duration distributions are fitted to power-laws using the powerlaw package [73].

## Supporting information

S1 Text Supplementary text, figures and extended modeling. We provide additional computations, numerical simulations, and an extended discussion of the model and its parametrizations.
(PDF)
