# Supplementary material for: Sampling effects and measurement overlap can bias the inference of neuronal avalanches
Source: PLoS Comput Biol. 2022 Nov 29;18(11):e1010678. doi: 10.1371/journal.pcbi.1010678 (PMC9733887; doi:10.1371/journal.pcbi.1010678)
Supplement: S1 Text — We provide additional computations, numerical simulations, and an extended discussion of the model and its parametrizations. (PDF) [file pcbi.1010678.s001.pdf]

Supplementary Information

Please note that a double-column version of this SI with working hyperlinks is available on arXiv:1910.09984.

1.1 Sampling bias remains under alternative topologies

The network topology used in the main paper is local: on average, each neuron is connected to its nearest $K = 10^3$ neighbors. It is of interest to check if alternative topologies can impact the distinguishability of the underlying dynamic state under coarse-sampling.

For that, we select two additional topologies. The first (”Orlandi”) mimics the growth process of a neuronal culture. In short, axons grow outward on a semiflexible path of limited length and have a given probability to form a synapse when they intersect the (circular) dendritic tree of another neuron. Thereby, this topology is local without requiring distance-dependent synaptic weights (refer to [1] for more details). The second (”Random”) implements a purely random connectivity, with each neuron being connected to $K = 10^3$ neurons. Note that this is an unrealistic setup as this topology is completely non-local.

We find that, under coarse-sampling, reverberating and critical dynamics remain indistinguishable with the alternative topologies (Fig *A*, left). Meanwhile, under sub-sampling, all dynamic states are clearly distinguishable for all topologies (Fig *A*, right).

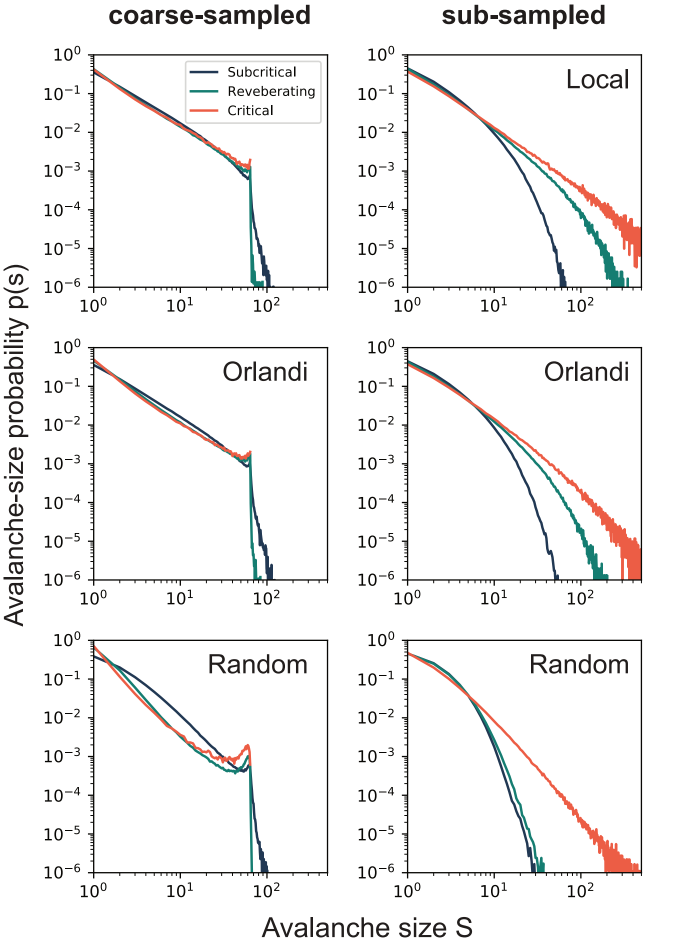

Fig *A*: Effect of alternative network topologies. Avalanche-size probability $p(S)$ from coarse-sampled activity (**left**) and sub-sampled activity (**right**) for subcritical, reverberating and critical dynamics. **Top**: results for the topology used in the main paper (”Local”). **Middle**: results for a topology that mimics culture growth [1] (”Orlandi”). **Bottom**: results for a random topology. Under coarse-sampling, reverberating and critical dynamics are indistinguishable with all topologies. Parameters: $d_{\mathrm{E}} = 400$ µm and $\Delta t = 8$ ms.

1.2 Influence of the electrode field-of-view

In the main paper we considered that the contribution of a spiking neuron to the electrode signal decays with distance d as $\sim 1/d$ (see Ref. [3, 4]). The precise way neuronal activity is recorded by extracellular electrodes depends on many factors such as neuronal morphology and the level of correlation between synapses [2, 4]. Because it is difficult to account for all relevant factors, we instead prioritized a simple, mechanistic approach.

In more detail, we are interested in the impact of a spike as a function of the distance to the electrode. Thus, two main contributions arise: first, the transmembrane currents along the pre-synaptic neuron and the currents at the synapses and post-synaptic neurons (which do not necessarily cause further spikes). This creates a distance-dependence of the LFP contribution that depends on the neuron's morphology and the connectivity profile. Second, the LFP signal has a distance-dependent decay depending on whether the source corresponds to an electric monopole ($1/d$) or dipole ($1/d^2$). The particular decay was found to depend, among other factors, on neuron type and morphology, and it effectively varies from $1/d$ near the soma to $1/d^2$ (and steeper) in the far-field limit [2–4]. Together, a more realistic LFP model would need to incorporate the connectivity profile of the neurons and the signal decay (the final effect of a neuron would be a convolution of both functions).

In our simplified model, we neglect neuron morphology (because our simple binary neurons only feature a single compartment) and the connectivity profile (because our topology is homogeneous and local). Thus, our implemented distance-dependence of the electrode signal merely serves an effective description that sensibly depends on the considered population.

As a sensitivity analysis for our effective description, we here study the impact of a varying electrode field of view (Fig *B*). An important detail that we neglect in the main manuscript is that the $1/d$ contribution does not extend into the far-field limit (beyond 100–1000 µm) [2, 3]. We implemented various different effective distance-dependencies, which are motivated by the work of Einevoll and colleagues, especially Fig 2D of Ref. [2]. Note however, that the reported shape function describes the LFP contribution as a function of a neuron receiving input, whereas, in our case, the potential describes the contribution of a spiking neuron.

In particular, we checked $\gamma = 2$ (Fig *B*, right column), which represents a very narrow electrodes' field of view. In

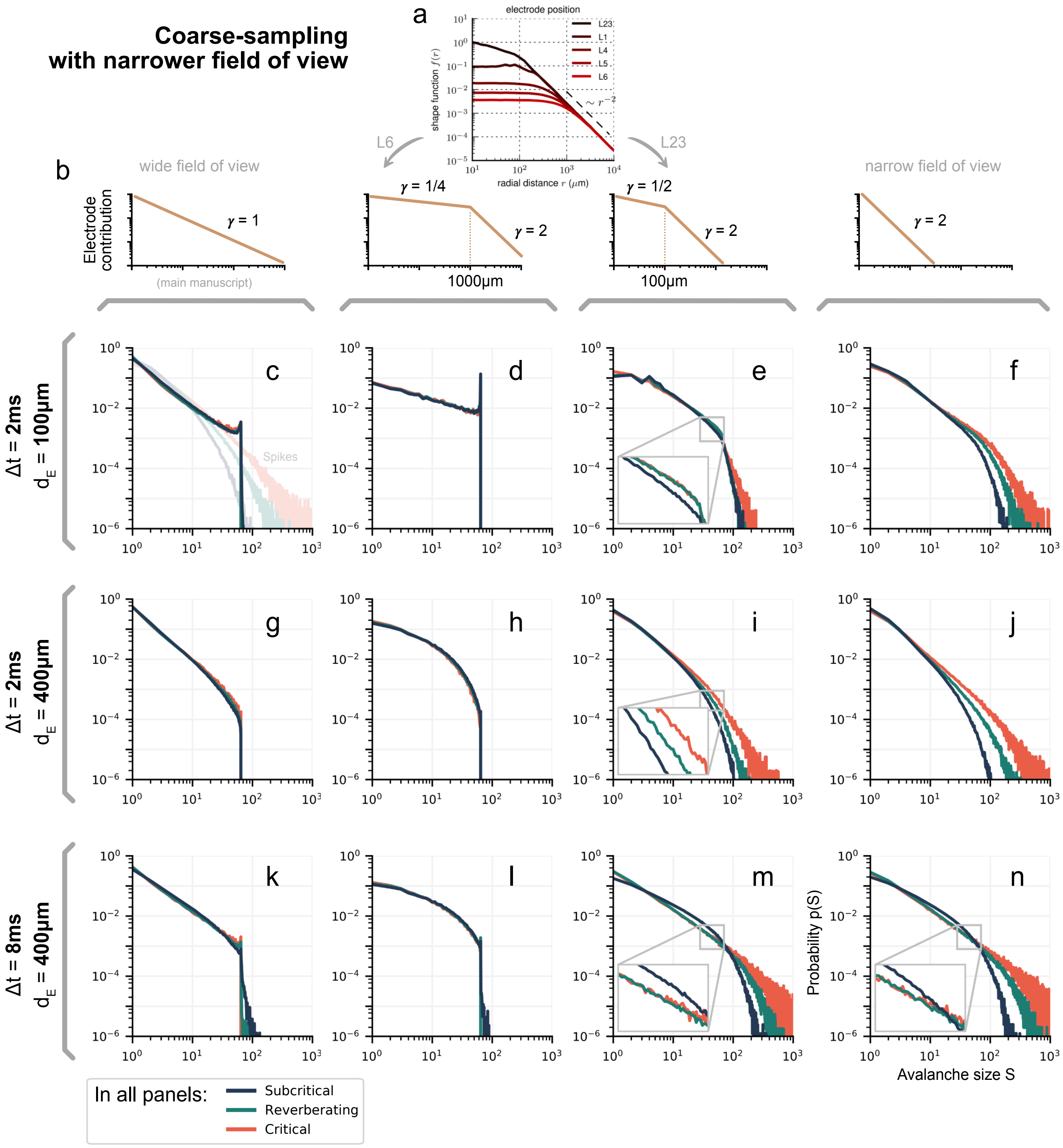

Fig *B*: Effect of changing the electrode contribution of a spiking neuron at distance d. a: Biophysically more plausible distance dependence of LFP, reproduced from [2]. **b:** Sketch of distance dependencies of tested effective electrode contribution that are *motivated* by (A). See accompanying text. Large decay exponents γ correspond to a narrow field of view of the electrodes (right column). When the transition from shallow to large exponents occurs at smaller d, electrodes can record fewer units and measurement overlap decreases. Eventually, distributions become distinguishable. As a notable side-effect, also the cut-off near $S = 64$ starts to vanish.

this case, avalanche-distributions become distinguishable, but the particular shape, cut-off and the amount of overlap between states again depends on the electrode distance and time-bin size.

We also checked contributions with a shallow exponent ($\gamma \leq 1$) near the electrode, which changes into a steep exponent ($\gamma = 2$) beyond a certain distance. For shallow exponents that reach far (transition at 1000 μm, Fig *B* d, h, l), the distributions of all considered states overlap, as in the main manuscript. Notably, the change in shape due to

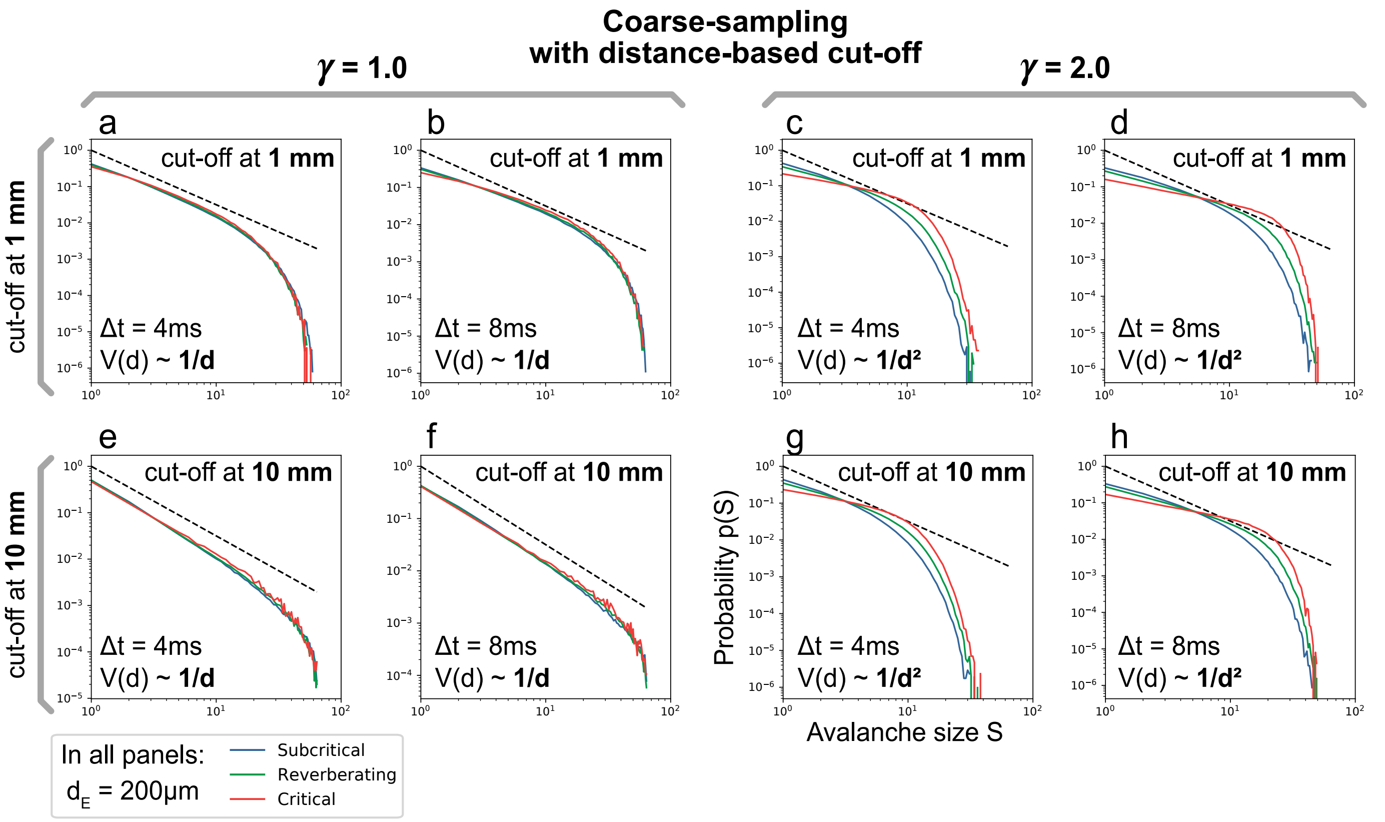

Fig *C*: Coarse-sampled avalanche-size distributions remain ambiguous under limited spatial reach. Results from the same model of the main paper, but with a hard cut-off at 1mm (top row) and 10mm (bottom row). Neurons farther away than the cut-off do not contribute to an electrode's signal. The effect of the cut-off stacks with the effect of the electrode decay-exponent γ. When $\gamma = 1$, dynamic states remain indistinguishable, independent from the cut-off. (For larger decay exponent states are distinguishable, cf. Fig *B*.) Notably, also for $\gamma = 1$, the shape of distributions is altered by the cut-off. As a guide to the eye, dashed lines indicate a power law with exponent -1.5. **Parameters:** $\gamma = 1$ (left half) and $\gamma = 2$ (right half), $d_{\mathrm{E}} = 200\,\mu\mathrm{m}$ in all panels.

changing the inter-electrode distance is even more severe than for $\gamma = 1$. For shallow exponents that do not reach far (transition at $100\,\mu\mathrm{m}$, Fig *B* e, i, m), distributions start to be distinguishable. However, in the usually considered range of avalanche sizes extending up to the number of electrodes ($S = 64$), reverberating and critical dynamics still tend to overlap (E, M). Only for short time bins ($\Delta t = 2\,\mathrm{ms}$) along large inter-electrode distances ($d_{\mathrm{E}} = 400\,\mu\mathrm{m}$) are the two states distinguishable (I).

Summarizing Fig *B*, we find that a main cause of collapsing avalanche distributions is a large electrodes' field of view. As the field of view becomes narrower, the relative contribution of the closest neurons to the electrode increases, and coarse-sampling becomes more similar to sub-sampling. The cut-off at $S \sim N_{\mathrm{E}}$ vanishes for steeper decays (large γ), and the different dynamic states become distinguishable.

For completeness, we performed further checks which follow the same reasoning. First, instead of changing exponents, we implemented a hard cut-off, beyond which neurons cannot contribute to an electrodes' signal (Fig *C*). We again found that, for cut-off values ($\geq 1\,\mathrm{mm}$), our main finding that avalanche distributions from different dynamic states are hardly distinguishable is unaltered. For short-range cut-off values that approach the distance between neurons ($d_{\mathrm{N}} = 50\,\mu\mathrm{m}$), distributions become distinguishable (not shown). This effect is similar to what we saw for increasing the decay exponent $\gamma \to 2.0$, where only individual neurons remain in the field of view of the electrode. In both theses cases, coarse-sampling starts to observe single-unit properties and becomes similar to the case of applying spike detection (here, sub-sampling).

Second, as our electrode potential is only an effective description and cannot be directly compared to Ref. [2], we additionally considered a hypothetical decay with exponent $\gamma = 1.5$ and repeated the analysis on different topologies (Figs. *H* and *I*). In all cases, $\gamma \geq 1.5$ causes the cut-off near $S = N_{\mathrm{E}}$ to vanish, and an increase of γ weakens the coarse-sampling effect. Note, however, that even with relatively narrow field of view, avalanche distribution from coarse-sampling still differ from those of spike analysis.

The above changes of the electrode model indicate that in real electrode recordings both effects are present, sub-sampling *and* coarse-sampling. In particular, the resulting avalanche distributions change on a continuous scale where our descriptions of coarse-sampling and sub-sampling are the extremes. Underlying dynamic states are better distinguishable when the sampling is "closer" to sampling single units instead of weighted averages — and when the measurement overlap that is characteristic to coarse-sampling

goes to zero.

Lastly, we want to caution about a peculiarity of $1/d^1$. From a geometric point of view, one has to consider how the number of neurons per volume element increases with distance from the electrode. In two dimensions, the number of neurons contained in a thin ring around an electrode scales $N \sim 2\pi d$. If $\gamma = 1$, the contributions of far-away neurons could be as strong as the contributions of close-by neurons, especially if activity is correlated.

In more detail, let the ring be of inner radius d and outer radius $d + \epsilon$, then its area is $A = 2\pi(d\epsilon + \epsilon^2)$. At a constant density ρ, the number of neurons in this ring is given by $N = \rho A$, so that, up to a constant, $N \sim 2\pi d\epsilon\rho$. Let $x_i = \{0, 1\}$ denote the state of a single neuron. Here, we assume that all neurons are uncorrelated and independent identically distributed random variables, with expectation value $\langle x \rangle = \mu$ and the same variance $\mathrm{Var}[x]$. Our electrode potential was modeled as

$$V = \sum_{i=1}^{N} x_i/d_i \,. \tag{1}$$

For every ring, we assume a constant $d_i = d$ for all neurons in the ring. Then, the expected potential for the ring is

$$\langle V(d) \rangle = \left\langle \sum_{i=1}^{N} x_i/d \right\rangle = \sum_{i=1}^{N} \langle x_i/d \rangle = N\mu/d \,. \tag{2}$$

Thus, when $N \sim 2\pi d\epsilon\rho$, then $\langle V(d) \rangle \sim \mu$. Indeed, this implies that the contributed potential of any of these rings is constant and that many neurons in a far-away ring can "contribute as much" as few local ones.

However, the variance of the potential per ring is not constant. For uncorrelated x, the variance of their sum is equal to the sum of their variances:

$$\mathrm{Var}[V(d)] = \mathrm{Var}\left[\sum_{i=1}^{N} x_i/d\right] = \sum_{i=1}^{N} \mathrm{Var}[x_i/d] \,. \tag{3}$$

With

$$\begin{aligned}\mathrm{Var}[x_i/d] &= \langle (x_i/d)^2 \rangle - \langle x_i/d \rangle^2 && (4)\\ &= \frac{1}{d^2}(\langle x_i^2 \rangle - \langle x_i \rangle^2) && (5)\\ &= \frac{1}{d^2}\mathrm{Var}[x_i] && (6)\end{aligned}$$

we see that

$$\mathrm{Var}[V(d)] = \frac{1}{d^2}\sum_{i=1}^{N} \mathrm{Var}[x_i] = \frac{N}{d^2}\mathrm{Var}[x] \,. \tag{7}$$

When again considering $N \sim 2\pi d\epsilon\rho$,

$$\mathrm{Var}[V(d)] \sim \mathrm{Var}[x]\epsilon\rho/d \,. \tag{8}$$

Hence, the standard deviation of the rings vanishes as $d \to \infty$; far away rings do contribute to the electrode, but they do not add to the variance of the signal. During the avalanche detection, the start or end of an avalanche is given by a threshold crossing. In a signal with more variance, more threshold crossing will occur, possibly leading to different avalanche statistics. As we showed, far-away neurons that are uncorrelated increase the mean, but they do not increase the variance, and, thus, do not lead to more (or less) threshold crossings. Notwithstanding, this reasoning only holds for uncorrelated neurons. If x_i are correlated, also far-away neurons could contribute to the variance. Although we think that is not the main cause for the coarse-sampling effect (cf. Fig *B*), we want to stress the limited range of electrodes for future work.

For a population of neurons receiving (un-) correlated synaptic input, the distance dependence of the LFP signal is studied in much more detail in Ref. [2]. Together with the above reasoning, this work highlights another possible source of sampling bias (which also affects our model electrodes): Because closer-to-critical dynamic states typically feature more correlated activity than subcritical states, the effective distance-dependence of an electrode is also affected by the dynamic state that is being recorded.

To conclude this section, we want to sketch an idealized experimental set-up: in order to determine criticality under coarse-sampling, the set-up should combine i) a large distance between electrodes d_{E}, ii) a narrow electrode field-of-view (large γ) and, ideally, iii) systems to calibrate with, which feature different dynamic states. This could potentially be used to qualitatively compare the distance to criticality between the systems. However, not only is this much more limited than what is possible with spike data [5–7], but the cut-off is a characteristic and ubiquitous feature commonly observed in experimental data of coarse-sampled recordings [8, 9]. This indicates that electrodes typically have a large field-of-view, and motivated our modeling assumption of $\gamma = 1$.

1.3 Neuron density

As the coarse sampling effect is sensitive to the field of view of electrodes, it may similarly depend on the amount of neurons any electrode captures, and, thus, the density of the neuron population. Hence, we performed a basic test of the robustness of our results with respect to the density of neurons (Fig *D*). To that end, we kept most parameters of the model as in the main manuscript: The culture extended over 4 cm substrate and neurons were hard-wired to their $\approx$ 1000 closest neighbors. A change in density impacts how far these neighbors are distributed, but we kept the effective projection range constant ($\sigma = 300\,\mu\mathrm{m}$), so that our "local" topology and the connectivity profile remain unchanged. We kept electrode contributions at $1/d$ and placed electrodes at a large distance (400 µm), so that every electrode samples many neurons and changes in density can become clear.

Compared to $\rho = 100/\,\mathrm{mm}^2$ of the main manuscript, we considered lower ($\rho = 25/\,\mathrm{mm}^2$) and higher densities ($\rho = 400/\,\mathrm{mm}^2$). Surprisingly, a change of neuron density only has a minor impact on the coarse sampling effect: Overall, the overlap of distributions and the cut-off remain for all considered densities (Fig *D*). Nonetheless, subtle differences are visible. Firstly, the slopes of the distributions seem to

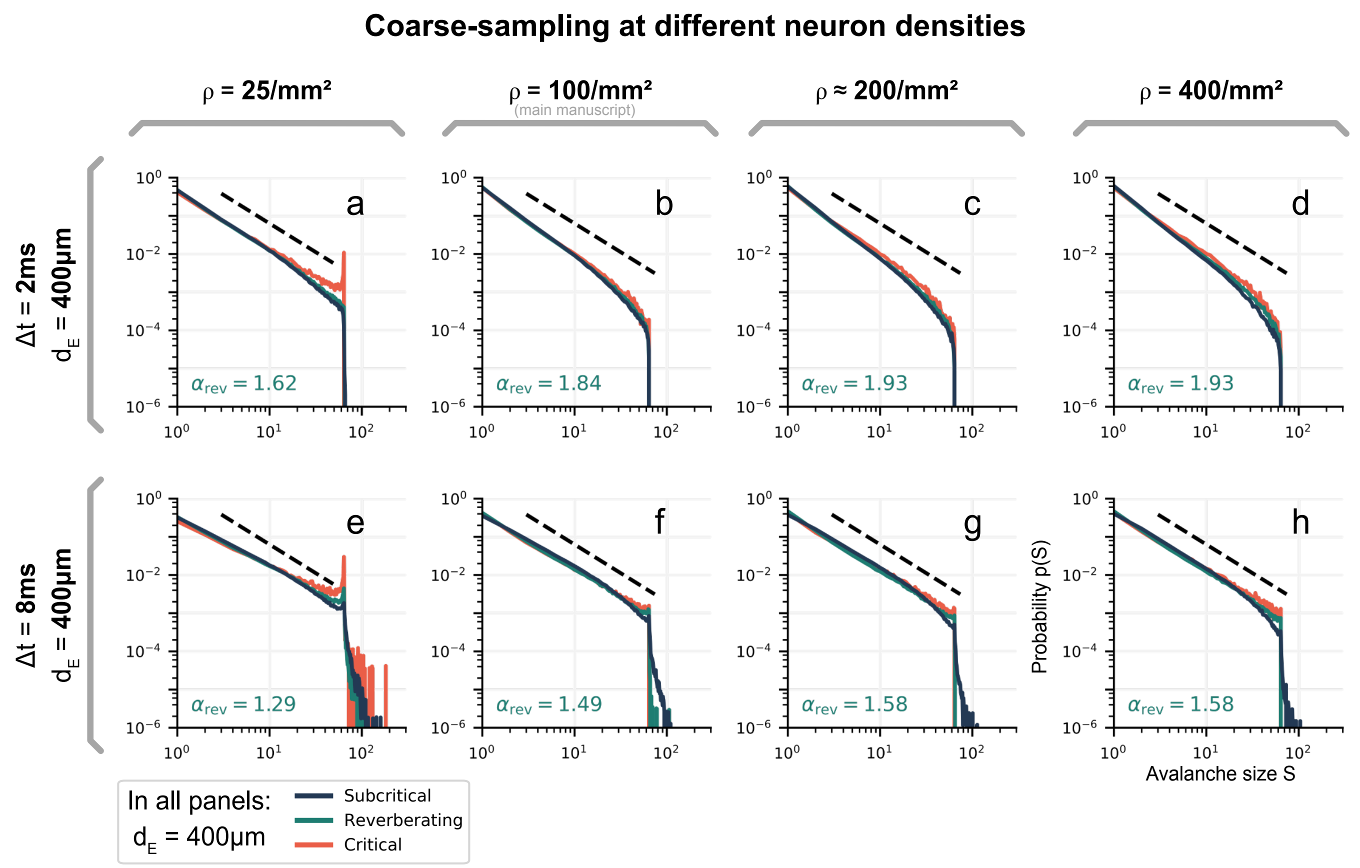

Fig *D*: Varying neuron densities. Simulations with parameters as in the main manuscript, with matching spatial extend of the substrate, connectivity profile of the neurons and field of view of the electrodes ($\gamma = 1$). As a guide to the eye, black dashed lines indicate $p(S) \sim S^{-1.5}$, and the fitted α for the reverberating state are indicated in the lower corner of every panel. Our main result that measurement overlap can render dynamic states indistinguishable is confirmed.

change, but this is shadowed by the dependence of the slope on the time-bin size. Secondly, for higher densities $\rho \geq 100/\,\mathrm{mm}^2$ distributions appear critical or slight subcritical, but for the low density, they also resemble super-critical distributions with a pronounced peak near the cut-off.

In conclusion, our main results concerning overlapping avalanche distributions for different dynamic states seem to be fairly invariant when increasing neuron density.

1.4 Low-pass filtering

A relevant question that we have not addressed in the main manuscript is how mechanisms that come into effect *before* the sampling hardware could impact avalanche statistics. Many studies are concerned with low-pass frequency filtering and how measurements of neuronal activity are affected. Such a temporal filtering may arise from the intrinsic neuronal morphology [2, 4, 10] or the surrounding extracellular tissue [11–13].

As a simple test that mimics natural low-pass filtering, we convolved the raw time series of every electrode with an exponentially decaying kernel (Fig *E*). Thus, the filtering was applied before the remaining analysis pipeline. The decay of the kernel (and the strength of the filtering) is parametrized through the decay time of the exponential. We considered decay times τ_f between 2 ms and 128 ms (ranging from 1 to 128 time steps of the simulation). The kernel was created using `scipy.signal.exponential` with a window size of 1000 time steps. The remaining analysis pipeline remained unchanged, and, in particular, included the frequency filtering to $0.1\,\mathrm{Hz} < f < 200\,\mathrm{Hz}$ that we assumed as part of the recording hardware.

Whereas the overlap of distributions largely remains when low-pass filtering is applied, the shape of the distributions depends on the strength of the filter. As a general trend, all distributions tend to form "super-critical" peaks as filtering becomes stronger. We associated these peaks with multiple electrodes picking up the same event (boosting the amount of large avalanches, up to the number of electrodes). This goes along with a decreased number of total avalanches that are detected (lower left corner in all panels). Note that the same raw time series with the same duration were used along every row of Fig *E*.

Together, this is consistent with the expecting "smoothing" of the raw time series due to low-pass filtering: Deflections of a time series around its mean get attenuated, and small excursions (at high frequency) become rare. Because these excursions potentially trigger the start of a new avalanche, fewer avalanches (or events) are detected when filtering becomes stronger. Intriguingly, the change of the

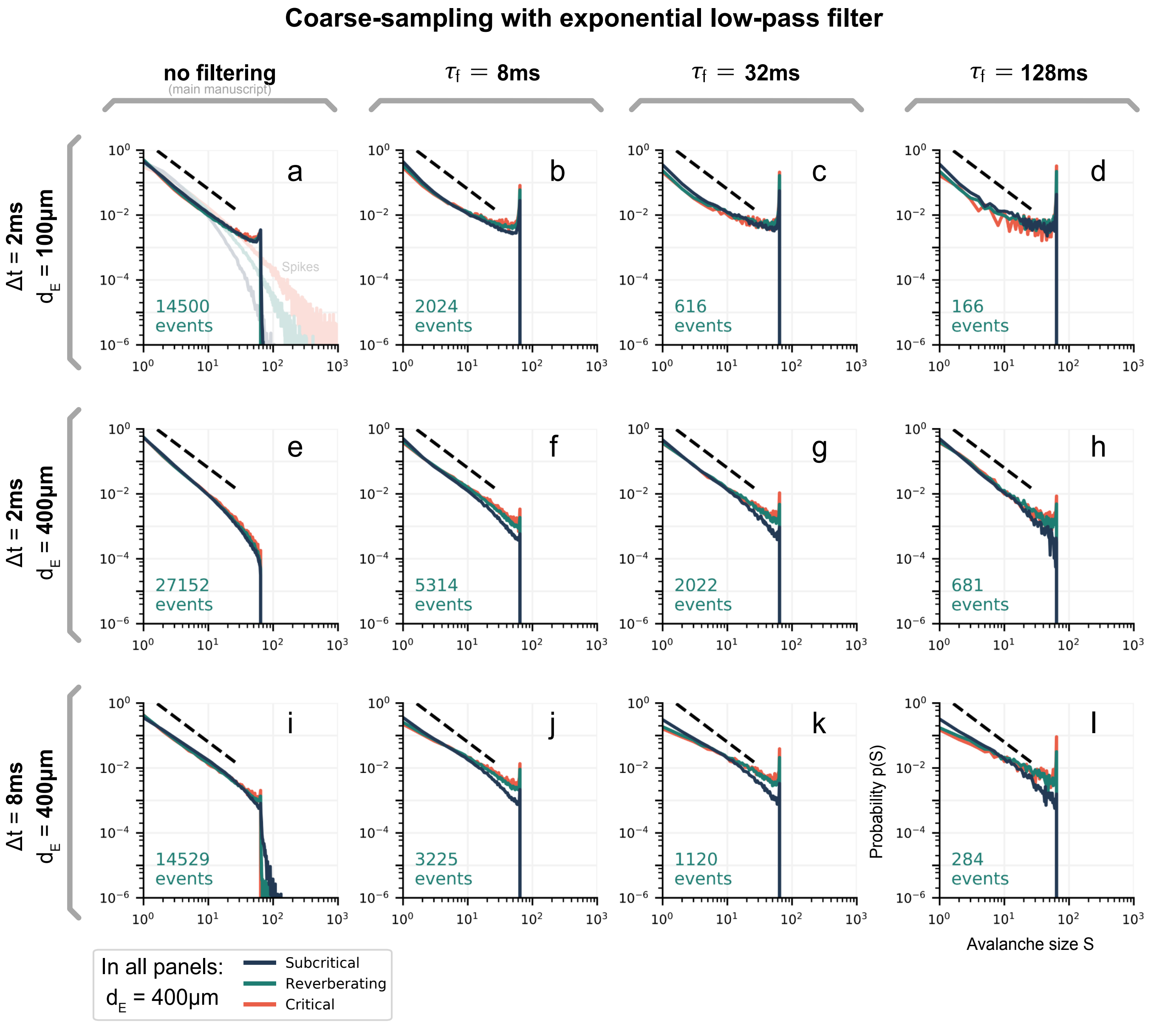

Fig *E*: Low-pass filtering using an exponential kernel. To mimic low-pass filtering as possibly caused by extracellular tissue or neuron morphology, an exponential kernel with decay time τ_{f} was applied to the raw signal before applying the analysis pipeline of the main manuscript (which includes frequency filtering to $0.1\,\mathrm{Hz} < f < 200\,\mathrm{Hz}$). As a guide to the eye, black dashed lines indicate $p(S) \sim S^{-1.5}$, and the number of events that contributed to the distribution for reverberating dynamics are indicated in the lower corner of every panel.

distribution shape due to filtering seems to affect critical and reverberating dynamics more severely than subcritical dynamics (especially visible in the bottom row).

However, note that the filtering employed here only serves as an example for a low-pass filter. Experimentally, power-spectra are often found to show $1/f^{\beta}$ scaling, with $0 < \beta < 4$, which limits the functional form a more realistic filtering kernel might have [12, 14–16].

1.5 Scaling laws may fail under coarse-sampling

The most used indication of criticality in neuronal dynamics is the avalanche-size distribution $p(S)$. However, at criticality, the *avalanche duration distribution* $p(D)$ and the *average avalanche size* for a given duration, $\langle S \rangle(D)$, should also follow power-laws, each with a respective *critical exponent* [17]:

$$p(S) \sim S^{-\alpha} \tag{9}$$

$$p(D) \sim D^{-\beta} \tag{10}$$

$$\langle S \rangle(D) \sim D^{\gamma} \tag{11}$$

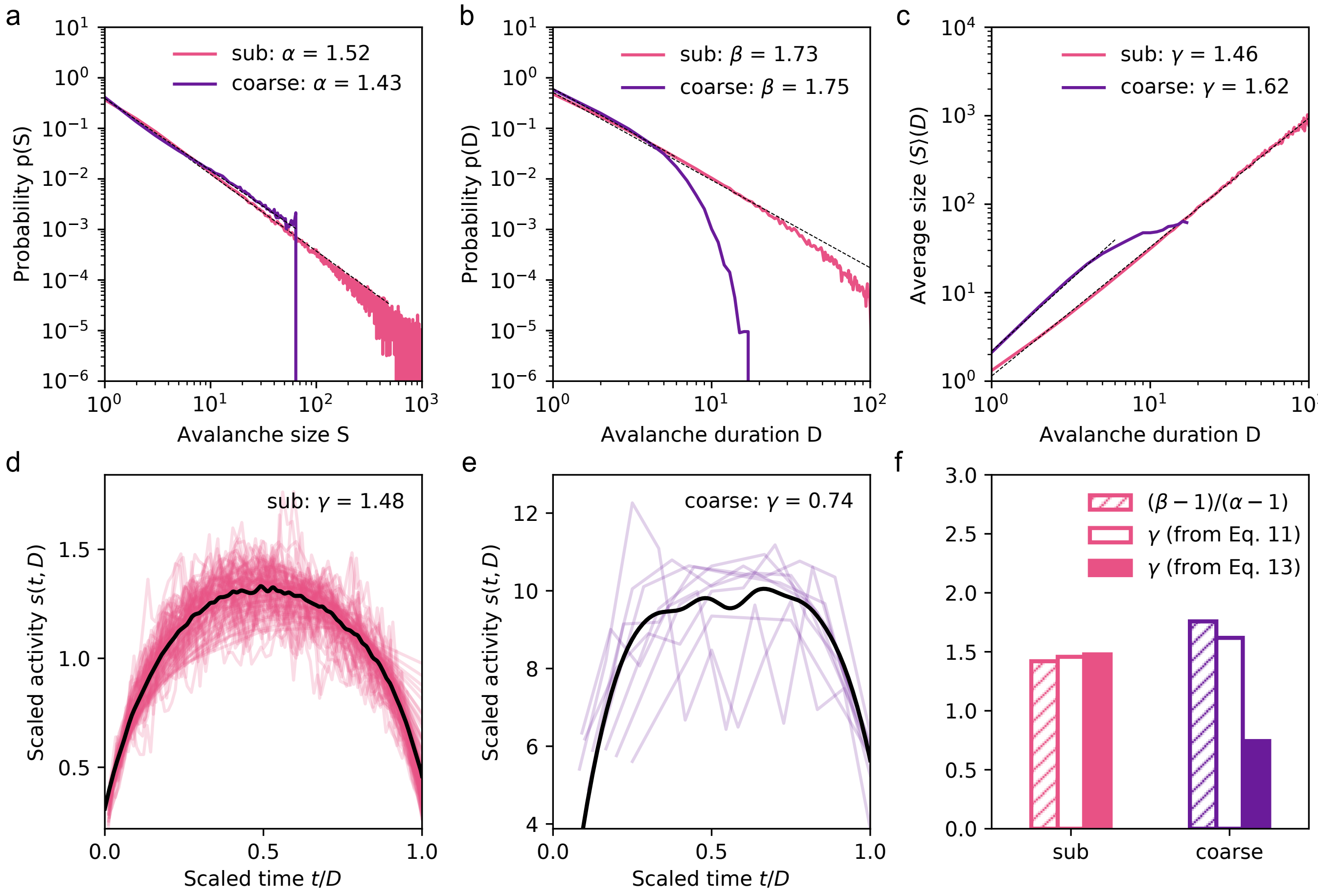

Fig *F*: Scaling laws of a system with critical dynamics under coarse- and sub-sampling. a–c: Avalanche-size distribution $p(S) \sim S^{-\alpha}$, avalanche-duration distribution $p(D) \sim D^{-\beta}$, and average size for a given duration $\langle S\rangle(D) \sim D^{\gamma}$, respectively, for sub-sampled ("sub") and coarse-sampled ("coarse") simulations. Distributions under sub-sampling easily span more than one order of magnitude, while coarse-sampled distributions suffer from an early cut-off (which hinders power-law fits). **d, e:** Shape collapse of $s(t, D) \sim D^{\gamma-1}\mathcal{F}(t/D)$ for sub-sampled and coarse-sampled data, respectively. Under coarse-sampling, the early duration cut-off results in few unique shapes for the collapse (corresponding to unique D-values). **f:** Comparison of the critical exponents obtained independently from Eqs. (11)–(13). Exponents are consistent only under sub-sampling. **Parameters:** $d_{\mathrm{E}} = 400\,\mu\mathrm{m}$ and $\Delta t = 8\,\mathrm{ms}$.

The exponents are related to one another by the scaling relationship*

$$\frac{\beta - 1}{\alpha - 1} = \gamma\,. \tag{12}$$

For a pure branching process — or any process in the mean-field directed percolation universality class [18, 19] — they take the values $\alpha = 3/2$, $\beta = 2$ and $\gamma = 2$.

Lastly, at criticality, avalanches of vastly different duration still have the same *average shape*: The activity $s(t, D)$ at any given time t (within the avalanche's lifetime D) is described by a universal scaling function $\mathcal{F}$, so that

$$s(t, D) \sim D^{\gamma-1}\mathcal{F}(t/D)\,. \tag{13}$$

In other words, changing $s(t, D) \to s(t, D)/D^{\gamma-1}$ and $t \to t/D$ should result in a data collapse for the average avalanche shapes of all durations.

The shape collapse of Eq. 13 is done following the algorithm described in [20]. Briefly, the avalanche profiles $s(t, D)$ of all avalanches with the same duration D are averaged, and the resulting curve is scaled to t/D and interpolated on 1000 points in the $[0, 1]$ range. Avalanches with $D < 4$, or with less than 20 realizations are removed. The chosen collapse exponent γ is the one that minimizes the error function:

$$E = \frac{\langle \mathrm{Var}(X_D/D^{\gamma-1})\rangle}{\Delta X^2} \tag{14}$$

where $X_D(t/D)$ is the interpolated average shape of avalanches with size D, and $\Delta X = \max_{t,D}(X_D/D^{\gamma-1}) - \min_{t,D}(X_D/D^{\gamma-1})$. The variance Var(.) is calculated over all valid D, and the mean $\langle . \rangle$ is taken over the scaled duration t/D. For interpolation and minimization we use the scipy [21] functions interpolate.InterpolatedUnivariateSpline and optimize.minimize, respectively.

From Eqs. (11)–(13), we have three independent ways to determine the exponent γ. Consistency between the three is a further test of criticality. However, to the best of our knowledge, experimental evidence with the full set of

*In this subsection, γ exclusively denotes the scaling exponent and the decay exponent (which is denoted with γ in the rest of the manuscript) equals 1 for all results presented here.

scaling laws is mainly observed under sub-sampling: from spikes of in vitro recordings † [23, 24], but see Ref. [22].

The absence of scaling laws in coarse-sampled data can be explained by how coarse-sampling biases the average shape: the cut-off in $p(S)$ near the number of electrodes $S = N_\mathrm{E}$ implies that $\langle S\rangle(D) < N_\mathrm{E}$. From Eq. (11) we have $D < N_\mathrm{E}^{1/\gamma}$. If $\gamma > 1$ the cut-off in $p(S)$ causes a much earlier cut-off in both $p(D)$ and $\langle S\rangle(D)$.

Given that experiments typically have $N_\mathrm{E} \sim 10^2$ electrodes, $p(D)$ of a pure branching process (with $\gamma = 2$) would span a power-law for less than one order of magnitude. However, the typical standard to reliably fit a power-law is at least two orders of magnitude [25]. While this is problematic under coarse-sampling (Fig 6), we have shown that the hard cut-off is not present under sub-sampling (Fig 7).

When we apply the independent measurements of γ to our model (with critical dynamics) under sub-sampling, we find consistent exponents for all measurements (Fig *F*f). Moreover, the exponents we find under sub-sampling are compatible with experimental values, e.g. $\gamma_\mathrm{exp} = 1.3 \pm 0.05$ reported in Ref. [23] and $1.3 \leq \gamma_\mathrm{exp} \leq 1.5$ reported in Ref. [24]. Notably, the exponents found in our model and experimentally differ from those expected for a pure branching process ($\gamma = 2$). While not the focus here, we believe this deviation to derive from topological properties of the network, which was also observed in Ref. [23]; distance-dependent weights of local topologies affect avalanche duration and size and yield different exponents than a branching process (which does not face any topological constraints).

When we apply the independent measurements of γ to our model (with critical dynamics) under coarse-sampling, exponents differ from measurement to measurement: The exponent obtained from the shape collapse ($\gamma \approx 0.74$) greatly differs from the other two ($\gamma \approx 1.74$, $\gamma \approx 1.62$), Fig *F*f. Moreover, the extremely short range available to fit $p(D)$ and $\langle S\rangle(D)$ with power-laws ($1 \leq D \leq 6$) makes the estimated exponents unreliable.

To conclude, the full set of critical exponents revealed criticality only under sub-sampling. Only in this case we observed both, a match between all the measurements of the exponent γ, and a power-law behavior extending over a range large enough to reliably fit them.

1.6 Coarse Graining the Ising Model

To demonstrate how general the impact of measurement overlap is, we study the two-dimensional Ising model. The Ising model is well understood and often serves as a textbook example for renormalization group (RG) theory in Statistical Physics [26]. In this framework, the system is *coarse grained* by merging multiple parts (spins) into one. An intuitive way to think of it is by zooming out of a photograph on a computer screen; a pixel can only show one color although there might be more details hidden underneath. Coarse graining is also known as the real-space block-spin renormalization and it can be used to assess criticality. Please note that *coarse graining* is different from *coarse-sampling.* Conventionally, coarse-graining perfectly tiles the space without any measurement-overlap (see Fig *G*).

The two-dimensional Ising model consists of $N = L^2$ spins with states $s_i = \pm 1$, arranged on a square lattice of length L. In its simplest form, it is given by the Hamiltonian $H(\vec{s}) = \sum_{\langle i,j\rangle} s_i s_j$, where $\langle i,j\rangle$ denotes all pairs of nearest neighboring spins. The probability of observing $\vec{s}$ is given by the Boltzmann distribution

$$P(\vec{s}, T) = \frac{1}{Z_T} e^{-H(\vec{s})/k_B T} \qquad (15)$$

where T is the temperature of the system, k_B is the Boltzmann constant (here, $k_\mathrm{B} = 1$) and Z_T is the partition function that normalizes the distribution. As the temperature $T \to T_c = 2/\ln(1+\sqrt{2})$, the system undergoes a second-order phase transition between a disordered spin configuration ($T > T_c$) and an ordered state of aligned spin orientations ($T < T_c$). Many observables diverge at $T = T_c$ for $L \to \infty$, such as correlation length, specific heat and susceptibility [18, 26].

We perform Monte Carlo simulations of the 2D Ising model using the massively parallel multicanonical method [27, 28]. The multicanonical method offers numerous advantages over conventional Monte Carlo approaches. For instance, instead of simulating at a single temperature, one simulation covers the whole energy space. High-precision canonical expectation values of observables are recovered for any desired temperature during a post-production step. Thereby, we obtain the normalized absolute magnetization as a function of temperature $m(T) = \frac{1}{N}|\sum_i s_i|$.

1.7 Block-Spin Transformation

Measurement overlap causes individual sources to contribute multiple times to a signal. For the Ising model, a similar process takes place when coarse graining is applied. In the process, spins are grouped into blocks of size $b \times b$, here $b = 4$ and every block only takes a single value. The value of each block can be obtained in different ways.

- Most commonly, the majority rule [26] is employed, where the block is assigned $+1$ (-1) if the majority of spins has value $+1$ (-1). In this case, the contribution of multiple sources is integrated. Hence we compare this rule to the effects observed when neuronal systems are coarse-sampled.

- Alternatively, one can use the decimation rule [26]. In this case, all except a single spin value within a block are discarded. The block value is assigned from the single spin that is kept. Hence we compare this rule to the effects observed when neuronal systems are sub-sampled.

This block-spin transformation rescales the number of spins by a factor of $1/b^2$, effectively reducing system size (which will cause finite-size effects). It is well known, that when studying the magnetization, the effective size of the compared systems (after rescaling) has to match.

†An exception can be found in Ref. [22], where scaling relations were found to hold in vivo. However, in this study, fluorescence imaging was coupled with very large time bins $\Delta t \geq 0.47\,\mathrm{s}$, the effect of which remains to be understood in full.

1.8 Overlap

To mimic the measurement overlap, we now introduce an overlap between the blocks of the Ising model coarse graining (Fig *G*). In the native block-spin transformation, blocks do not overlap. Then, in terms of spins, the linear distance d between two blocks matches the block size $b = d = 4$ (Fig *G*a). When the distance between blocks is smaller than the block size, $d < b$ (Fig *G*b), measurement overlap is created, while when $d > b$ parts of the system are not sampled. Clearly, the changes that such an overlap will cause on rescaled observables should depend on the rule used to perform the block-spin transformation.

Here, we look at combinations of block size $b = 4$ with distance between blocks of $d = 2$, $d = 4$ and $d = 8$. In order to preserve the effective system size ($L = 16$), we thus perform simulations for $L = 32$, $L = 64$ and $L = 128$, respectively.

Using the majority rule and no overlap — which is the default real-space renormalization-group approach — the procedure moves m away from $m(T_c)$ (Fig *G*c, $d = b$): For $T < T_c$, m is increased; For $T > T_c$, m is decreased. Ordinarily, T_c can be obtained by finding the crossing of m between an unblocked ($L = 16$) and a blocked ($L = 64$, $b = 4$) system — only at T_c is the measured m invariant under block rescaling transformations.

1.9 Majority Rule "coarse"

What is the impact of the overlap for the majority rule? For increasing overlap ($d < b$), the crossing occurs at $T > T_c$ (Fig *G*c). This is because sharing spins increases the correlations between blocks (pairwise and higher-order), making it more likely that the rescaled spins point into the same direction. In other words, it biases the measurement of m towards order, increasing our estimated critical temperature.

For absent overlap ($d > b$), only every other block is measured. This decorrelates the spins near the borders of each block and, therefore, decreases the correlation between blocks. As a consequence, the spin orientation of the blocked system moves towards disorder, decreasing the measured magnetization m.

1.10 Decimation Rule "sub"

If instead of the majority rule the decimation rule is used, the blocking procedure does not alter the correlation between spins before and after the transformation (Fig *G*d). As a consequence, the magnetization remains unaltered in general. However, in the disordered phase, we still notice a systematic deviation from the unblocked system (with $L = 64$). This deviation can be fully attributed to finite-size effects: The distribution of realizable magnetizations in the disordered phase follows a Gaussian with mean zero and variance proportional to the (effective) number of spins. Due to the definition of the magnetization with absolute value, the expectation value of the magnetization for $T \to \infty$ is determined by the (effective) system size.

As was the case when sub-sampling neuronal systems, the increase in correlation that ultimately leads to biased observables is caused by integrating weighted contributions from various sources. This is not the case when the decimation rule is applied. Note that the impact of different block-transformation rules on $m(T)$ will not hold for all other canonical observables such as the energy $E(T)$ [26].

References

1. Orlandi, J. G. *et al.* Noise focusing and the emergence of coherent activity in neuronal cultures. *Nature Physics* **9,** 582–590 (2013).
2. Linden, H. *et al.* Modeling the spatial reach of the LFP. *Neuron* **72,** 859–872 (2011).
3. Pettersen, K. H. & Einevoll, G. T. Amplitude Variability and Extracellular Low-Pass Filtering of Neuronal Spikes. *Biophysical Journal* **94,** 784–802 (2008).
4. Einevoll, G. T. *et al.* Modelling and analysis of local field potentials for studying the function of cortical circuits. *Nature Reviews Neuroscience* **14,** 770–785 (2013).
5. Wilting, J. & Priesemann, V. Inferring collective dynamical states from widely unobserved systems. *Nature Communications* **9,** 2325 (2018).
6. Wilting, J. & Priesemann, V. Between Perfectly Critical and Fully Irregular: A Reverberating Model Captures and Predicts Cortical Spike Propagation. *Cerebral Cortex* **29,** 2759–2770 (2019).
7. Levina, A. & Priesemann, V. Subsampling scaling. *Nature Communications* **8,** 15140 (2017).
8. Beggs, J. M. & Plenz, D. Neuronal Avalanches in Neocortical Circuits. *Journal of Neuroscience* **23,** 11167–11177 (2003).
9. Gireesh, E. D. & Plenz, D. Neuronal avalanches organize as nested theta- and beta/gamma-oscillations during development of cortical layer 2/3. *Proceedings of the National Academy of Sciences* **105,** 7576–7581 (2008).
10. Buzsáki, G. *et al.* The origin of extracellular fields and currents — EEG, ECoG, LFP and spikes. *Nature Reviews Neuroscience* **13,** 407–420 (2012).
11. Gabriel, S. *et al.* The dielectric properties of biological tissues: II. Measurements in the frequency range 10Hz to 20GHz. *Physics in Medicine and Biology* **41,** 2251–2269 (1996).
12. Bédard, C. *et al.* Does the 1/f Frequency Scaling of Brain Signals Reflect Self-Organized Critical States? *Physical Review Letters* **97,** 118102 (2006).
13. Bédard, C. & Destexhe, A. Macroscopic models of local field potentials and the apparent 1/f noise in brain activity. *Biophysical Journal* **96,** 2589–2603 (2009).
14. Miller, K. J. *et al.* Power-law scaling in the brain surface electric potential. *PLoS Computational Biology* **5** (2009).
15. Milstein, J. *et al.* Neuronal shot noise and Brownian 1/f2 behavior in the local field potential. *PloS one* **4,** e4338 (2009).
16. Donoghue, T. *et al.* Parameterizing Neural Power Spectra into Periodic and Aperiodic Components. *Nat Neurosci* **23,** 1655–1665 (2020).

Table *A*: Compilation of experimental findings of neuronal avalanches [29]. For cultures, "region" corresponds to the brain region neurons were extracted from. Range of $p(S)$ was either given in the text, or estimated visually. Exponents with "$\approx$" are not explicitly fitted but instead visually compared. Notably, most studies reporting power-law distributions in-vivo built on coarse-sampled measures, or imaging (in which case the effect of the low sampling-frequency on avalanche distributions is not clear). We found only one reference in the literature where in-vivo spiking data were used *and* power-law distributions occurred, although over a very limited range [30].

authors	technique	region	system	exponent α	range of $p(S)$
Beggs *et al.*, 2003 [8]	LFP	cortex	culture	≈ 1.5	1-60
Gireesh *et al.*, 2008 [9]	LFP	cortex	culture	1.53 ± 0.02	1-32
Petermann *et al.*, 2009 [31]	LFP	cortex	monkey	≈ 1.5	1-32
Klaus *et al.*, 2011 [32]	LFP	cortex	monkey	≈ 1.5	1-96
Priesemann *et al.*, 2013 [33]	LFP	varied	human	1.58 ± 0.06	1-50
Shew *et al.*, 2015 [34]	LFP	cortex (visual)	turtle	1.6-2.6	3-200
Clawson *et al.*, 2017 [35]	LFP	cortex (visual)	turtle	1.8 ± 0.3	2-500
Meisel *et al.*, 2013 [36]	MEEG	whole head	human	≈ 1.5	1-27
Palva *et al.*, 2013 [37]	MEEG	whole head	human	1.31	1-66
Shriki *et al.*, 2013 [38]	MEEG	whole head	human	≈ 1.5	1-100
Arviv *et al.*, 2015 [39]	MEEG	whole head	human	1.50	1-100
Scott *et al.*, 2014 [40]	imaging	cortex	rat	≈ 1.5	1-1000
Bellay *et al.*, 2015 [41]	imaging	cortex	rat	1.63 ± 0.13	0.01-10
Fagerholm *et al.*, 2015 [42]	imaging	cortex	rat	≈ 1.5	1-1000
Yaghoubi *et al.*, 2018 [43]	imaging	hippocampus	culture	1.65 ± 0.1	1-500
Ponce-Alvarez *et al.*, 2018 [22]	imaging	whole brain	zebrafish	2.01 ± 0.03	1-10000
Bocaccio *et al.*, 2019 [44]	imaging	whole head	human	≈ 2	1-1000
Friedman *et al.*, 2012 [23]	spikes	cortex	culture	1.7	1-30
Massobrio *et al.*, 2015 [45]	spikes	cortex	culture	1.48-1.52	1-500
Hahn *et al.*, 2017 [30]	spikes	cortex (visual)	monkey	1.58 ± 0.03	1-20
Levina *et al.*, 2017 [7]	spikes	cortex	culture	≈ 2	1-5900
Kanders *et al.*, 2020 [24]	spikes	hippocampus	culture	2.18 ± 0.05	1-60

17. Sethna, J. P. *et al.* Crackling noise. *Nature* **410,** 242–250 (2001).

18. Sethna, J. P. *Statistical Mechanics: Entropy, Order Parameters, and Complexity* 1st (Oxford University Press, New York, 2006).

19. Janssen, H. K. & Täuber, U. C. The field theory approach to percolation processes. *Annals of Physics* **315,** 147–192 (2005).

20. Marshall, N. *et al.* Analysis of Power Laws, Shape Collapses, and Neural Complexity: New Techniques and MATLAB Support via the NCC Toolbox. *Frontiers in Physiology* **7,** 1–18 (2016).

21. Virtanen, P. *et al.* SciPy 1.0: fundamental algorithms for scientific computing in Python. *Nature methods* (2020).

22. Ponce-Alvarez, A. *et al.* Whole-Brain Neuronal Activity Displays Crackling Noise Dynamics. *Neuron* **100,** 1446–1459 (2018).

23. Friedman, N. *et al.* Universal critical dynamics in high resolution neuronal avalanche data. *Physical Review Letters* **108,** 1–5 (2012).

24. Kanders, K. *et al.* Fingerprints of a second order critical line in developing neural networks. *Communications Physics* **3,** 13 (2020).

25. Stumpf, M. P. H. & Porter, M. A. Critical Truths About Power Laws. *Science* **335,** 665–666 (2012).

26. Newman, M. E. J. & G. T. Barkema. *Monte Carlo Methods in Statistical Physics* 1st, 490 (Oxford University Press, New York, 1999).

27. Zierenberg, J. *et al.* Scaling properties of a parallel implementation of the multicanonical algorithm. *Computer Physics Communications* **184,** 1155–1160 (2013).

28. Gross, J. *et al.* Massively parallel multicanonical simulations. *Computer Physics Communications* **224,** 387–395 (2018).

29. Neto, J. P. *Criticality and Sampling in Neural Networks* PhD thesis (University of Göttingen, 2020).

30. Hahn, G. *et al.* Spontaneous cortical activity is transiently poised close to criticality. *PLOS Computational Biology* **13,** e1005543 (2017).

31. Petermann, T. *et al.* Spontaneous cortical activity in awake monkeys composed of neuronal avalanches. *Proceedings of the National Academy of Sciences* **106,** 15921–15926 (2009).

32. Klaus, A. *et al.* Statistical analyses support power law distributions found in neuronal avalanches. *PLoS ONE* **6** (2011).

33. Priesemann, V. *et al.* Neuronal Avalanches Differ from Wakefulness to Deep Sleep – Evidence from Intracranial Depth Recordings in Humans. *PLoS Computational Biology* **9,** e1002985 (2013).

34. Shew, W. L. *et al.* Adaptation to sensory input tunes visual cortex to criticality. *Nature Physics* **11,** 659–663 (2015).

35. Clawson, W. P. *et al.* Adaptation towards scale-free dynamics improves cortical stimulus discrimination at the cost of reduced detection. *PLOS Computational Biology* **13,** e1005574 (2017).

36. Meisel, C. *et al.* Fading Signatures of Critical Brain Dynamics during Sustained Wakefulness in Humans. *Journal of Neuroscience* **33,** 17363–17372 (2013).

37. Palva, J. M. *et al.* Neuronal long-range temporal correlations and avalanche dynamics are correlated with behavioral scaling laws. *Proceedings of the National Academy of Sciences* **110,** 3585–3590 (2013).

38. Shriki, O. *et al.* Neuronal Avalanches in the Resting MEG of the Human Brain. *Journal of Neuroscience* **33,** 7079–7090 (2013).

39. Arviv, O. *et al.* Near-Critical Dynamics in Stimulus-Evoked Activity of the Human Brain and Its Relation to Spontaneous Resting-State Activity. *Journal of Neuroscience* **35,** 13927–13942 (2015).

40. Scott, G. *et al.* Voltage Imaging of Waking Mouse Cortex Reveals Emergence of Critical Neuronal Dynamics. *Journal of Neuroscience* **34,** 16611–16620 (2014).

41. Bellay, T. *et al.* Irregular spiking of pyramidal neurons organizes as scale-invariant neuronal avalanches in the awake state. *eLife* **4,** 1–25 (2015).

42. Fagerholm, E. D. *et al.* Cascades and Cognitive State: Focused Attention Incurs Subcritical Dynamics. *Journal of Neuroscience* **35,** 4626–4634 (2015).

43. Yaghoubi, M. *et al.* Neuronal avalanche dynamics indicates different universality classes in neuronal cultures. *Scientific Reports* **8,** 3417 (2018).

44. Bocaccio, H. *et al.* The avalanche-like behaviour of large-scale haemodynamic activity from wakefulness to deep sleep. *Journal of the Royal Society Interface* **16** (2019).

45. Massobrio, P. *et al.* Self-organized criticality in cortical assemblies occurs in concurrent scale-free and small-world networks. *Scientific Reports* **5,** 1–16 (2015).

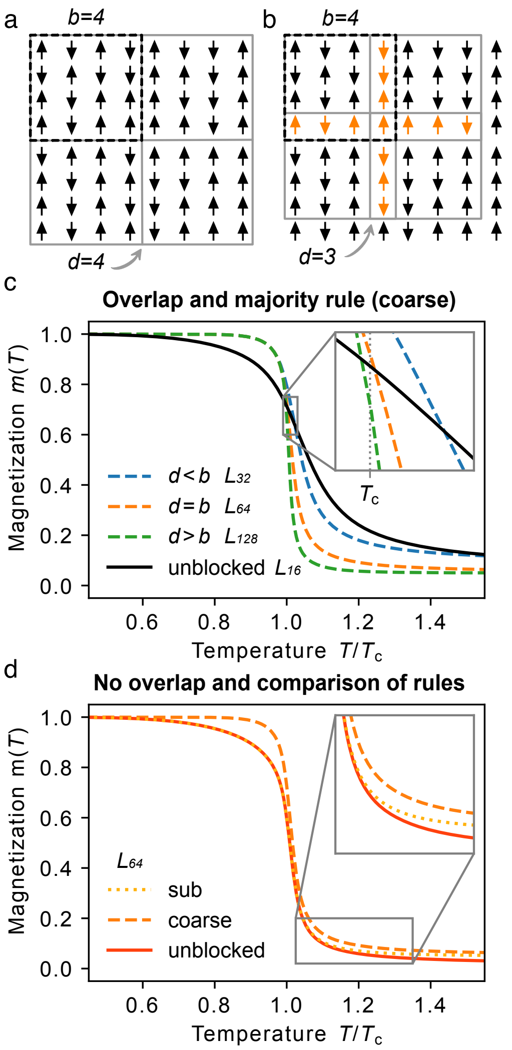

Fig *G*: Coarse graining the Ising model. a: Representation of the standard coarse graining where block size matches the distance between blocks ($d = b = 4$). No overlap is created. **b:** Coarse graining with block size $b = 4$ and a distance between blocks of $d = 3$. Overlapping spins (orange) are shared by two or more blocks. **c:** With the "coarse" majority rule, overlap impacts the spontaneous magnetization $m(T)$. Only the crossing between the unblocked ($L = 16$) and non-overlapping blocked system ($d = b$, $L = 64$) happens at $T = T_C$, as would be expected. Intriguingly, the overlap ($d < b$, $L = 32$) pushes the system towards higher magnetization where spins appear more aligned. On the other hand, the absence of overlap ($d > b$, $L = 128$) causes smaller magnetization where spins appear more random. (Note that, in order to avoid finite-size effects, the target size after coarse graining has to match, here $L = 16$. Consequently, depending on the ratio between d and b, simulations have different system sizes.) **d:** Comparison between the fully-sampled, unblocked system and blocked systems using the majority rule ("coarse") and the decimation rule ("sub") for $d = b = 4$. All simulations and curves for $L = 64$. In the ordered, low-temperature phase, the sub curve matches the fully sampled system. Only for the high-temperature phase deviations occur due to finite-size effects (the magnetization for $T \to \infty$ approaches the value expected for the rescaled $L = 16$ system). The coarse curve is systematically biased towards more ordered states.

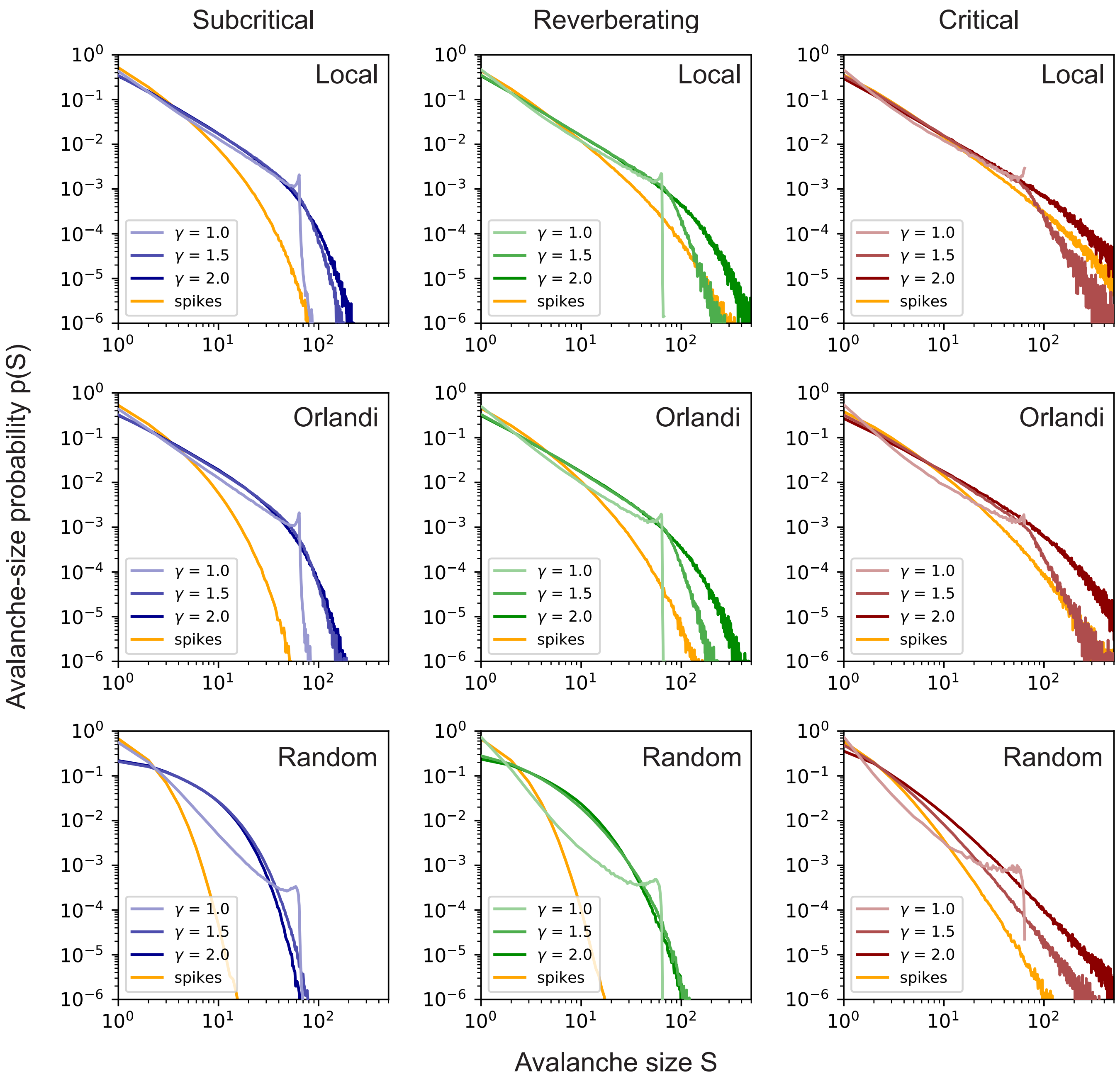

Fig *H*: Effect of changing the electrode contribution $\sim 1/d^{\gamma}$ of a spiking neuron at distance d, for different network topologies and $d_{\mathrm{E}} = 200\,\mu\mathrm{m}$. Dynamic states are Subcritical (**left**), Reverberating (**center**) and Critical (**right**). Topologies are Local (**top**), Orlandi (**middle**) and Random (**bottom**). Local corresponds to the topology used in the main paper, Orlandi corresponds to the model described in [1], and Random corresponds to a completely random topology. Increasing γ (decreasing electrode FOV) results in a loss of the cut-off for $p(S) \sim N_{\mathrm{E}}$ as the coarse-sampling becomes more spike-like. Bin-size for all distributions is $\varDelta t = 4\,\mathrm{ms}$.

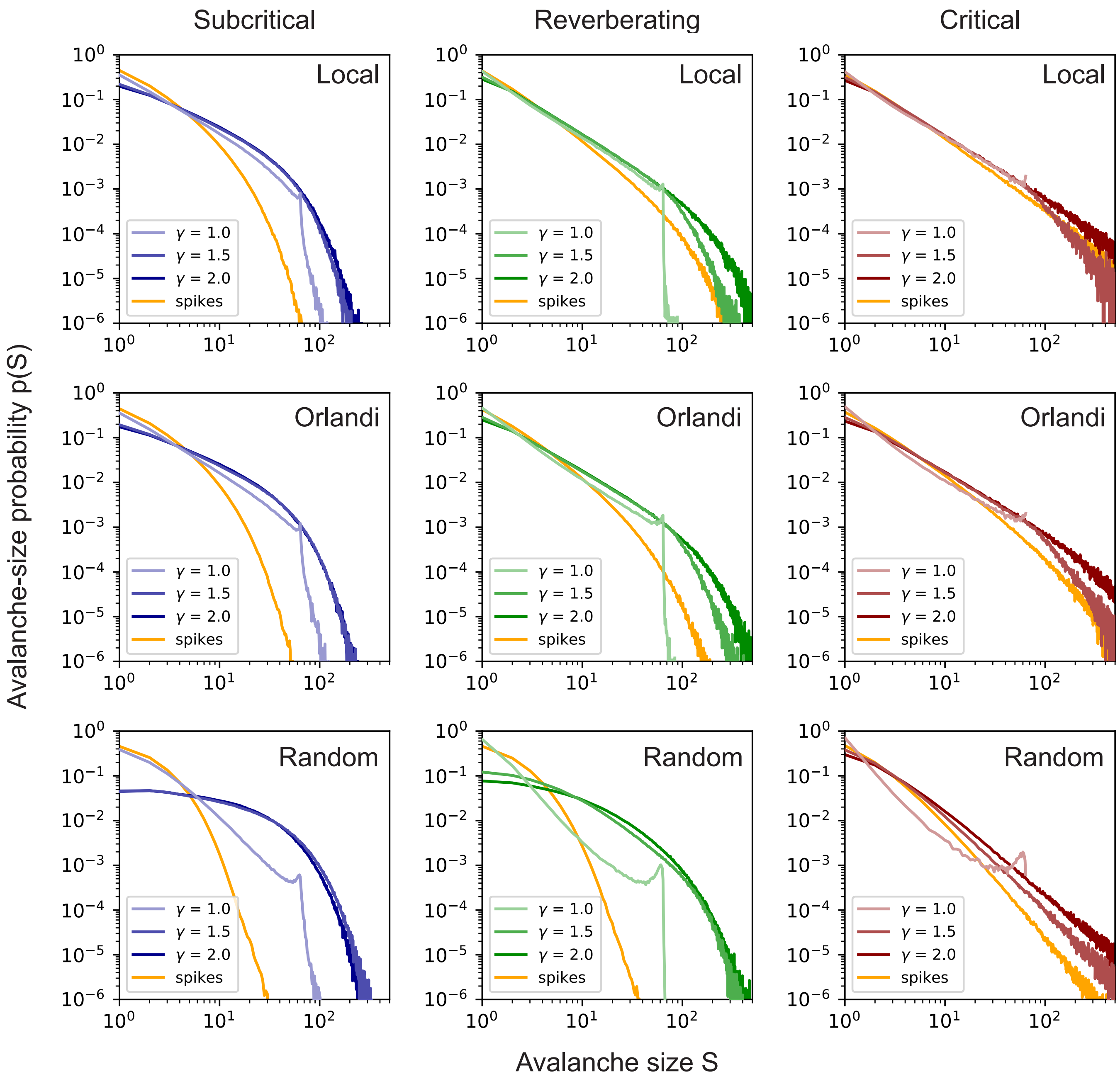

Fig *I*: Effect of changing the electrode contribution $\sim 1/d^{\gamma}$ of a spiking neuron at distance d, for different network topologies and $d_{\mathrm{E}} = 400\,\mu\mathrm{m}$. Dynamic states are Subcritical (**left**), Reverberating (**center**) and Critical (**right**). Topologies are Local (**top**), Orlandi (**middle**) and Random (**bottom**). Local corresponds to the topology used in the main paper, Orlandi corresponds to the model described in [1], and Random corresponds to a completely random topology. Increasing γ (decreasing electrode FOV) results in a loss of the cut-off for $p(S) \sim N_{\mathrm{E}}$ as the coarse-sampling becomes more spike-like. Bin-size for all distributions is $\Delta t = 8\,\mathrm{ms}$.

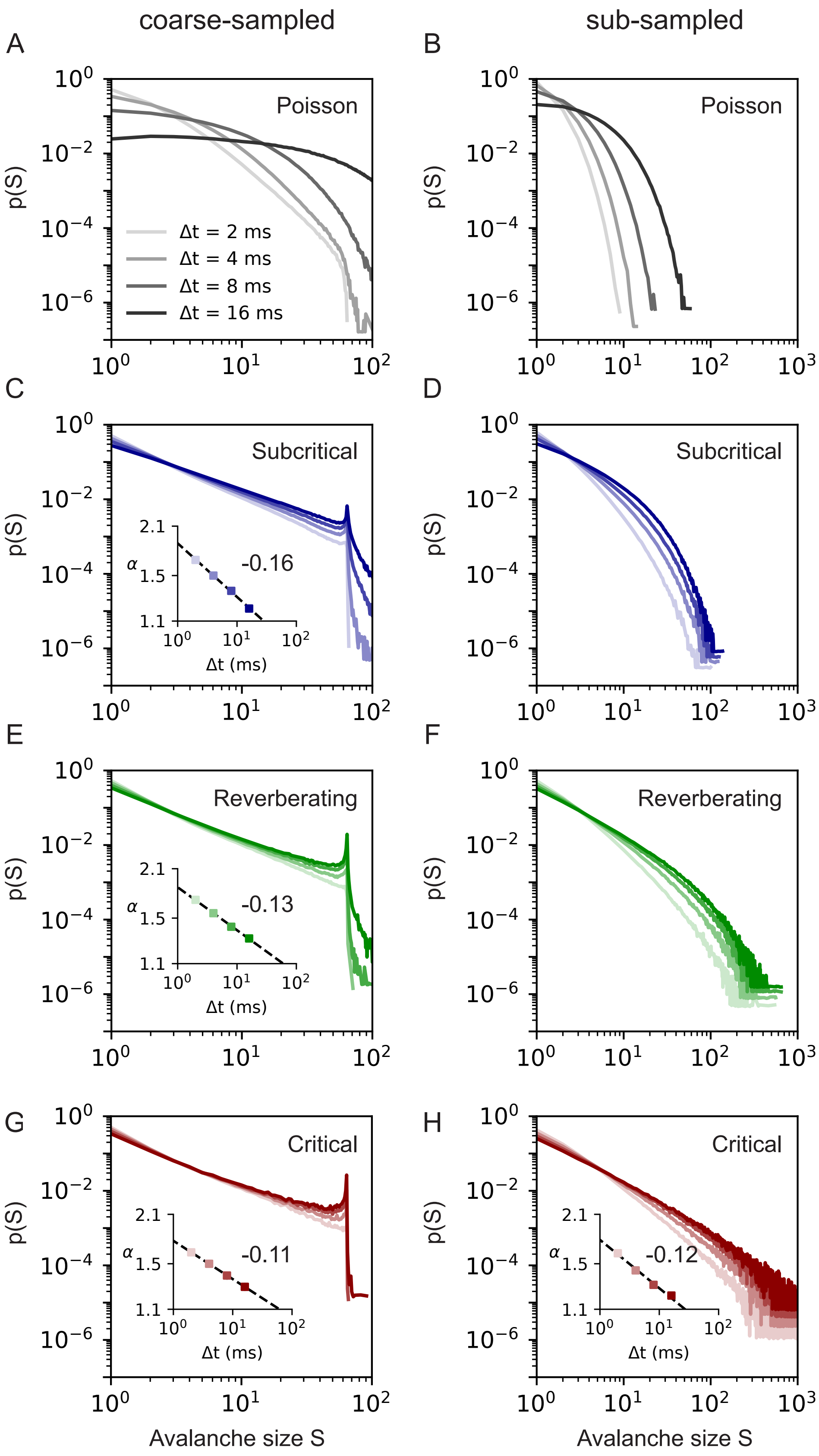

Fig *J*: Avalanche-size distributions $p(S)$ dependence on time-bin size Δt for $d_{\mathrm{E}} = 200\,\mu\mathrm{m}$. Coarse-sampled (**left**) and sub-sampled (**right**) results from an array of 64 virtual electrodes with time bin sizes between $2\,\mathrm{ms} \leq \Delta t \leq 16\,\mathrm{ms}$. Dynamics states are Poisson (**a-b**), Subcritical (**c-d**), Reverberating (**e-f**) and Critical (**g-h**). Distributions are fitted to $p(S) \sim S^{\alpha}$. **Insets:** Dependence of α on Δt, fitted as $\alpha \sim \Delta t^{-\beta}$. Fit values are shown in Table. 3.
